# Synthesis and Evaluation of Novel 2*H*-Benzo[*e*]-[1,2,4]thiadiazine 1,1-Dioxide Derivatives as PI3Kδ Inhibitors

**DOI:** 10.3390/molecules24234299

**Published:** 2019-11-25

**Authors:** Ya-Ping Gong, Long-Qian Tang, Tong-Shen Liu, Zhao-Peng Liu

**Affiliations:** Department of Medicinal Chemistry, Key Laboratory of Chemical Biology (Ministry of Education), School of Pharmaceutical Sciences, Shandong University, Jinan 250012, China; 201614474@mail.sdu.edu.cn (Y.-P.G.); lqtang.student@sina.com (L.-Q.T.); jshrlts@163.com (T.-S.L.)

**Keywords:** PI3Ks, PI3Kδ inhibitors, 2*H*-benzo[*e*][1,2,4]thiadiazine 1,1-dioxide, anticancer, anticancer agents

## Abstract

In previous work, we applied the rotation-limiting strategy and introduced a substituent at the 3-position of the pyrazolo [3,4-*d*]pyrimidin-4-amine as the affinity element to interact with the deeper hydrophobic pocket, discovered a series of novel quinazolinones as potent PI3Kδ inhibitors. Among them, the indole derivative **3** is one of the most selective PI3Kδ inhibitors and the 3,4-dimethoxyphenyl derivative **4** is a potent and selective dual PI3Kδ/γ inhibitor. In this study, we replaced the carbonyl group in the quinazolinone core with a sulfonyl group, designed a series of novel 2*H*-benzo[*e*][1,2,4]thiadiazine 1,1-dioxide derivatives as PI3Kδ inhibitors. After the reduction of nitro group in *N*-(2,6-dimethylphenyl)-2-nitrobenzenesulfonamide **5** and *N*-(2,6-dimethylphenyl)-2-nitro-5-fluorobenzenesulfonamide **6**, the resulting 2-aminobenzenesulfonamides were reacted with trimethyl orthoacetate to give the 3-methyl-2*H*-benzo[*e*][1,2,4]thiadiazine 1,1-dioxide derivatives. After bromination of the 3-methyl group, the nucleophilic substitution with the 3-iodo-1*H*-pyrazolo[3,4-*d*]pyrimidin-4-amine provided the respective iodide derivatives, which were further reacted with a series of arylboronic acids via Suzuki coupling to furnish the 2*H*-benzo[*e*][1,2,4]thiadiazine 1,1-dioxide derivatives **15a**–**J** and **16a**–**d**. In agreement with the quinazolinone derivatives, the introduction of a 5-indolyl or 3,4-dimethoxyphenyl at the affinity pocket generated the most potent analogues **15a** and **15b** with the IC_50_ values of 217 to 266 nM, respectively. In comparison with the quinazolinone lead compounds **3** and **4**, these 2*H*-benzo[*e*][1,2,4]thiadiazine 1,1-dioxide derivatives exhibited much decreased PI3Kδ inhibitory potency, but maintained the high selectivity over other PI3K isoforms. Unlike the quinazolinone lead compound **4** that was a dual PI3Kδ/γ inhibitor, the benzthiadiazine 1,1-dioxide **15b** with the same 3,4-dimethoxyphenyl moiety was more than 21-fold selective over PI3Kγ. Moreover, the introducing of a fluorine atom at the 7-position of the 2*H*-benzo[*e*][1,2,4]thiadiazine 1,1-dioxide core, in general, was not favored for the PI3Kδ inhibitory activity. In agreement with their high PI3Kδ selectivity, **15a** and **15b** significantly inhibited the SU-DHL-6 cell proliferation.

## 1. Introduction

**Figure 1:**
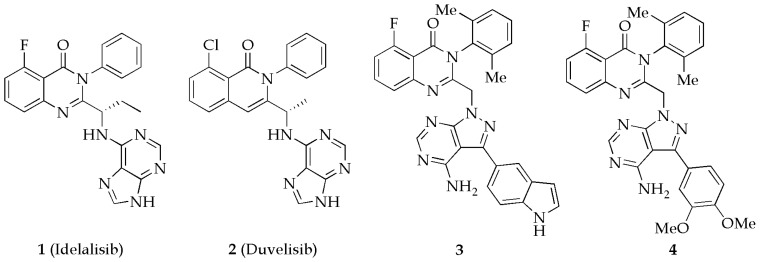
Selective PI3Kδ and dual PI3Kδ/γ inhibitors.

Phosphoinositide 3-kinases (PI3Ks) are a family of lipid kinases that regulate numerous biological functions, including cell growth, proliferation, differentiation, motility, and intracellular trafficking, through the phosphorylation of the phosphatidylinositol 4,5-bisphosphate (PIP2) to generate the lipid second messenger phosphatidylinositol 3,4,5-trisphosphate (PIP3) [1,2,3]. There are three classes of the PI3K enzyme, of which class I PI3Ks are the mostly studied and further divided into subgroups IA (PI3Kα, PI3Kβ, and PI3Kδ) and IB (PI3Kγ) based on the signaling pathways and the regulatory proteins to which they bind [4,5]. The class IA PI3K isoforms mediate the signal transduction from receptor tyrosine kinases [6], while the IB isoform PI3Kγ is principally activated by G-protein coupled receptors [7]. PI3Kα and PI3Kβ are ubiquitously expressed, while PI3Kδ and PI3Kγ are dominantly expressed in leukocytes [8,9,10]. All the class I PI3K isoforms are implicated in cancer [11,12,13,14,15,16]. There are also mounting evidences that support a therapeutic role for inhibition of PI3Kα in diabetes [17,18], PI3Kβ in thrombosis [19,20], PI3Kδ and PI3Kγ in both rheumatoid arthritis and asthma [21,22,23,24], PI3Kδ in activated PI3Kδ syndrome (APDS) [25,26,27], and PI3Kγ in idiopathic pulmonary fibrosis [28]. Therefore, the development of PI3K isoform selective inhibitors is a promising therapeutic strategy for the treatment of these PI3Ks-related diseases. The selective PI3Kδ inhibitor idelalisib (Figure 1) is approved by FDA for follicular lymphoma (FL) and small lymphocytic lymphoma (SLL) and for chronic lymphocytic leukemia (CLL) in combination with rituximab [29,30]. The dual PI3Kδ/γ inhibitor duvelisib (Figure 1) is approved for adult patients with relapsed or refractory CLL or SLL, and relapsed or refractory FL after at least two prior systemic therapies [31]. However, in the clinical application of idelalisib, infectious and autoimmune toxicities were observed, and the unique toxicities are associated with inhibition of different isoforms of the PI3K enzyme [32]. To improve the isoform selectivity of the quinazolinone-based PI3Kδ inhibitors, in previous work, we introduced a pyrazolo [3,4-*d*]pyrimidin-4-amine moiety as the hinge region binding group, a substituent at the 3-position of the pyrazolo[3,4-*d*]pyrimidine core as the affinity element to interact with the deeper hydrophobic pocket, and a 2,6-dimethylphenyl to limit the free rotation of the 3-phenyl in idelalisib, discovered the indole derivative **3** as one of the most selective PI3Kδ inhibitors (IC_50_ = 8.6 nM) with more than 3630-fold, 390-fold and 40-fold selective for PI3Kδ over PI3Kα, β and γ, and the 3,4-dimethoxyphenyl derivative **4** as a potent and selective dual PI3Kδ/γ inhibitor (IC_50_ = 8.4 nM for PI3Kδ, IC_50_ = 62 nM for PI3Kγ) with more than 1400-fold, 820-fold selective for PI3Kδ over PI3Kα and PI3Kβ [33]. Considering the importance of sulfonamides in drug discovery [34,35,36], we replaced the carbonyl group in the quinazolinone core, and reported here the synthesis and preliminary evaluation of 2*H*-benzo[*e*][1,2,4]thiadiazine 1,1-dioxide derivatives as PI3Kδ inhibitors.

## 2. Results and Discussion

### 2.1. Chemistry

**Scheme 1:**
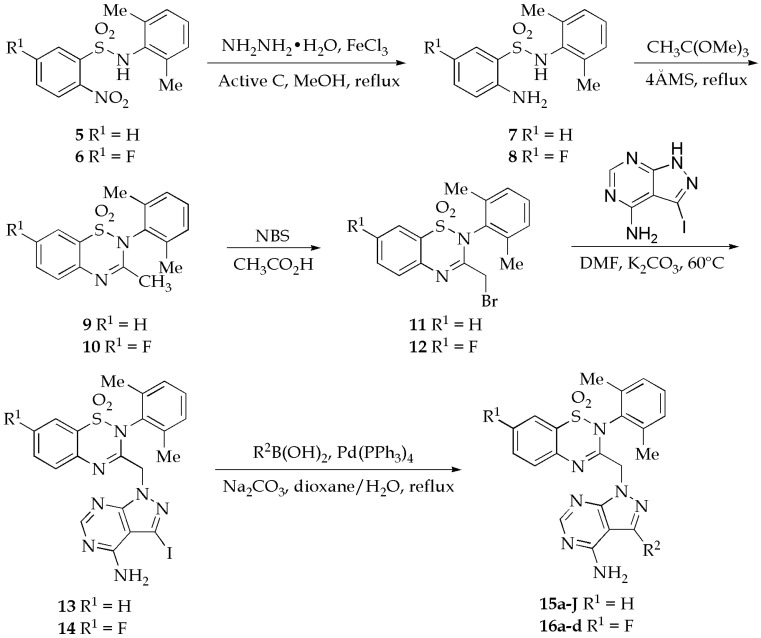
Synthesis of 2*H*-benzo[*e*][1,2,4]thiadiazine 1,1-dioxide derivatives.

All the new 2*H*-benzo[*e*][1,2,4]thiadiazine 1,1-dioxide derivatives were prepared following a general synthetic route shown in Scheme 1. The 2-nitrobenzene-1-sulfonamides **5** and **6** were readily prepared according to the reported method by the reaction of 2-nitrobenzene-1-sulfonyl chloride or 5-fluoro-2-nitrobenzene-1-sulfonyl chloride with 2,6-dimethylbenzenamine in methanol and water solution in the presence of CH_3_COONa under refluxing conditions [37]. Reduction of the nitro group to amine was carried out using hydrazine monohydrate in the presence of ferric chloride and activated charcoal in methanol under reflux conditions in excellent yields (95% and 99%). The resulting 2-aminobenzenesulfonamides **7** and **8** were reacted with trimethyl orthoacetate to give the corresponding 3-methyl-2*H*-benzo[*e*][1,2,4]thiadiazine 1,1-dioxide derivatives **9** and **10** in 51% and 40%, respectively. In the bromination of the allyl methyl group using *N*-bromosuccinimide (NBS), the main compounds were found to be the dibrominated products. Therefore, compounds **9** and **10** were reacted with only 0.5 equivalent of NBS in glacial acetic acid to give the monobrominated derivatives **11** and **12** in moderate yields (79% and 72% based on NBS). Nucleophilic substitution of **11** and **12** with 3-iodo-1*H*-pyrazolo[3,4-*d*]pyrimidin-4-amine, which was readily prepared from 5-amino-1*H*-pyrazole-4-carbonitrile in two steps by the known procedures [38], resulted in the iodides **13** and **14** in 86 and 58% yields, respectively. Finally, the incorporation of the affinity elements was achieved through the Suzuki coupling of **13** and **14** with the appropriate boronic acid in dioxane and water catalyzed by Pd(PPh_3_)_4_ under refluxing conditions, and the target 2*H*-benzo[*e*][1,2,4]thiadiazine 1,1-dioxide derivatives **15a**–**J** and **16a**–**d** were obtained in 34–91% and 51–84% yields. The structures of these 2*H*-benzo[*e*][1,2,4]thiadiazine 1,1-dioxide derivatives were characterized by ^1^H-nuclear magnetic resonance (NMR) and ^13^C-NMR (please refer to the Supplementary Materials).

### 2.2. PI3Kδ Inhibitory Activity and Isoform Selectivity

Compounds **15a**–**J** were first tested for their inhibitory activity against PI3Kδ using the ADP-Glo luminescent assay [39], using a pan-PI3K inhibitor PI-103 as a positive control [40]. As shown in Table 1, the substitution at the 3-position of the pyrazolo[3,4-d]pyrimidine with 5-indolyl or 3,4-dimethoxyphenyl led to the relative potent analogues **15a** and **15b** with IC_50_ values of 217 to 266 nM, respectively. The 6-methoxypyridin-3-yl derivative **15d** exhibited moderate PI3Kδ inhibitory activity (IC_50_ = 498 nM), whereas the 3-fluoro-4-methoxyphenyl analogue **15c** only had marginal activity. In comparison with **15b**, the substitution of the 3,4-dimethoxypheny group for 2,3-dihydrobenzo[*b*][1,4]dioxin-6-yl (**15e**), benzo[*d*][1,3]dioxol-5-yl (**15f**), 4-methoxyphenyl (**15g**), 3-methoxyphenyl (**15h**), 4-(trifluoromethoxy)phenyl (**15i**), and phenyl (**15j**) was not tolerated, indicating the subtle requirements at the affinity pocket of PI3Kδ. 

In order to increase the inhibitory activity of compound **15a**–**d**, a fluorine atom was introduced at the 7-position of the 2*H*-benzo[*e*][1,2,4]thiadiazine 1,1-dioxide, and compounds **16a**–**d** were prepared and evaluated for their PI3Kδ inhibitory activity. In comparison with **15a**, the fluorinated compound **16a** lost its activity (Table 1), and compounds **16b** and **16d** showed almost 2-fold decrease in potency. In contrast, compound **16c** with a 3-fluoro-4-methoxyphenyl moiety at the affinity pocket showed a slight increase in potency.

Compared with the leading quinazolinone derivatives **3** and **4**, these 2*H*-benzo[*e*][1,2,4]-thiadiazine 1,1-dioxide derivatives **15a**–**j** and **16a**–**d** showed much decreased PI3Kδ inhibitory activity. However, the most potent derivatives **15a** and **15b** proved to be selective PI3Kδ inhibitors (Table 2). The indole derivative **15a** showed significantly lower potency against other three isoforms of class I PI3K and was more than 140-fold selective for PI3Kδ over PI3Kα, β and γ. The 3,4-dimethoxyphenyl derivative **15b** was more than 60-fold, 90-fold and 20-fold selective for PI3Kδ over PI3Kα, PI3Kβ, and PI3Kγ, respectively. In comparison with the lead **4**, **15b** was more selective over PI3Kγ (21-fold vs. 7-fold).

### 2.3. SU-DHL-6 Cell Growth Inhibitory Activity 

The selective PI3Kδ inhibitors **15a** and **15b** were further evaluated for their antiproliferative activity against human B-cell SU-DHL-6. **15a** and **15b** significantly inhibited SU-DHL-6 cell proliferation with the GI_50_ of 2.13 and 2.50 μM, respectively (Table 2), which were in consistent with their PI3Kδ inhibitory potency.

### 2.4. Molecular Modeling Study

**Figure 2:**
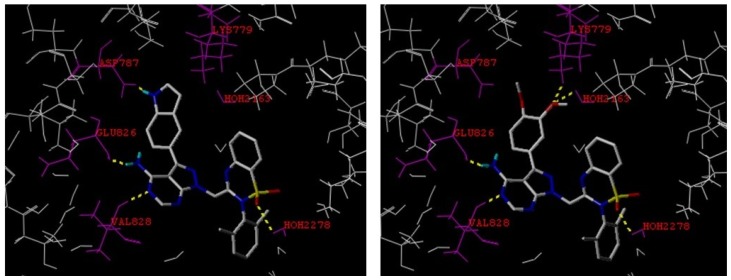
Molecular docking studies of **15a** (left) and **15b** (right).

Molecular docking studies were conducted on the new discovered selective PI3Kδ inhibitors **15a** and **15b**. As shown in Figure 2, the pyrazolo[3,4-d]pyrimidine portion in both compounds **15a** and **15b** forms hydrogen bonds with Glu826 and Val828 in the hinge region. 

Both inhibitors bind to the PI3Kδ isoform in an ‘induced fit’ conformation in which the 2*H*-benzo[*e*][1,2,4]thiadiazine 1,1-dioxide moiety is sandwiched between Trp760 and Met752 as these residues move apart to create the specificity pocket. The indol-5-yl (**15a**) in the specificity pocket forms an additional hydrogen bond with Asp787. Like the carbonyl oxygen in **3** and **4**, one sulfonyl oxygen in both **15a** and **15b** acts as hydrogen bond acceptor from the H_2_O2278. In compound **15b**, only the 3-methoxy group forms hydrogen bonding with Lys779, while in lead **4**, the 3-methoxy interacts with Tyr813 and Asp911, the 4-methoxy interacts with Lys779 [33]. These differences may contribute its less potency against PI3Kδ than **4**. In comparison with **3** and **4**, the lack of a 8-fluorine at the 2*H*-benzo[*e*][1,2,4]thiadiazine 1,1-dioxide core in **15a** and **15b** may also be related to their lower PI3Kδ inhibitory activity.

## 3. Materials and Methods

### 3.1. General Chemical Experimental Procedures

^1^H- and ^13^C-NMR spectra were recorded on a Bruker-600 NMR spectrometer (Brucker Co., Ltd., Zurich, Switzerland). All spectra were recorded at room temperature for DMSO or CDCl_3_ solutions. High resolution mass spectra (HRMS) were obtained on a 6520 QTOF instrument (Agilent Technologies Inc.*,* Santa Clara, CA, USA by electrospray ionization (ESI). Melting points were determined on an X-6 micromelting point apparatus (Beijing Tech. Co., Ltd., Beijing, China) without corrections. Column chromatography was performed on silica gel (200–300 mesh). All reactions involving oxygen- or moisture sensitive compounds were carried out under a dry N_2_ atmosphere using anhydrous solvents. Unless otherwise noted, reagents were added by syringe. 

*2-Amino-N-(2,6-dimethylphenyl)benzenesulfonamide* (**7**) 

To a stirred solution of *N*-(2,6-dimethylphenyl)-2-nitrobenzenesulfonamide (**5**, 19.4 g, 63.3 mmol) in methanol (200 mL), ferric chloride (5.1 g, 19 mmol) and activated charcoal (6.5 g) was added and refluxed for 30 min. 80% Hydrazine monohydrate (31.7 g, 633 mmol) was then added dropwise and refluxed for 5 h. After filtration, the filtrate was concentrated and the residue was dissolved in EtOAc (200 mL), washed with brine, dried over anhydrous Na_2_SO_4_. After filtration and evaporation, the residue was purified by silica gel chromatography (EtOAc/hexane = 1:3) to give **7** (16.5 g, 95%) as a white solid, m.p. 144–146 °C; lit. [41] m.p. 144–145 °C.

*2-Amino-N-(2,6-dimethylphenyl)-5-fluorobenzenesulfonamide* (**8**)

According to the procedures described for the synthesis of **7**, compound **8** were obtained as a colorless solid (16 g) in 99% yield, m.p. 185–186 °C; ^1^H-NMR (DMSO-d_6_) δ 9.44 (s, 1H, SO_2_NH), 7.21 (td, *J* = 9.0, 3.0 Hz, 1H, Ar-H), 7.07 (td, *J* = 8.4, 1.5 Hz, 1H, Ar-H), 7.01 (d, *J* = 7.8 Hz, 2H, Ar-H), 6.97 (dd, *J* = 8.4, 3.0 Hz, 1H, Ar-H), 6.85 (dd, *J* = 9.0, 4.8 Hz, 1H, Ar-H), 5.85 (s, 2H, NH_2_), 2.04 (s, 6H, 2,6-(CH_3_)_2_); MS (ESI) calcd. for C_1__4_H_14_FN_2_O_2_S [M − H]^−^: 293.1, found: 293.3.

*2-(2,6-Dimethylphenyl)-3-methy-2H-benzo[e][1,2,4]thiadiazine 1,1-dioxide* (**9**) 

A mixture of **7** (5 g, 18.1 mmol), trimethyl orthoacetate (50 mL) and 4Ă molecular sieve (10 g) was refluxed for 10 h. After cooling to room temp., the mixture was concentrated and the residue was dissolved in EtOAc (200 mL), washed with brine, dried over anhydrous Na_2_SO_4_. After filtration and evaporation, the residue was purified by silica gel chromatography (EtOAc/hexane = 1:5) to give **9** (2.8 g, 51%) as a white solid, m.p. 162–163 °C; ^1^H-NMR (CDCl_3_) δ 7.89 (d, *J* = 7.8 Hz, 1H, Ar-H), 7.70 (td, *J* = 8.4, 1.2 Hz, 1H, Ar-H), 7.60 (d, *J* = 8.4 Hz, 1H, Ar-H), 7.47 (td, *J* = 7.8, 1.2 Hz, 1H, Ar-H), 7.21 (t, *J* = 7.2 Hz, 1H, Ar-H), 7.18 (d, *J* = 7.8 Hz, 2H, Ar-H), 2.22 (s, 6H, 2,6-(CH_3_)_2_), 2.11 (s, 3H, 3-CH_3_); ^13^C-NMR (CDCl_3_) δ 154.34, 142.55, 138.58, 133.51, 132.59, 129.99, 129.27, 127.65, 127.23, 126.90, 121.01, 23.38, 18.62; MS (ESI) *m/z* calcd. for C_16_H_17_N_2_O_2_S [M + H]^+^ 301.1, found 301.0.

*2-(2,6-Dimethylphenyl)-7-fluoro-3-methy-2H-benzo[e][1,2,4]thiadiazine1,1-dioxide* (**10**) 

According to the procedures described for the synthesis of **9**, compound **10** were obtained as a colorless solid (6.9 g) in 40% yield, m.p. 150–151 °C; ^1^H-NMR (DMSO-d_6_) δ 7.89 (dt, *J* = 7.2, 1.5 Hz, 1H, Ar-H), 7.73 (dd, *J* = 7.2, 1.2 Hz, 2H, Ar-H), 7.36 (t, *J* = 7.2 Hz, 1H, Ar-H), 7.29 (d, *J* = 7.8 Hz, 2H, Ar-H), 2.13 (s, 6H, 2,6-(CH_3_)_2_), 2.06 (s, 3H, 3-CH_3_); ^13^C-NMR (DMSO-d_6_) δ 160.28 (d, *J*_C–F_ = 249.2 Hz), 154.02, 139.26 (d, *J*_C–F_ = 3.0 Hz), 138.59, 132.62, 130.88 (d, *J*_C–F_ = 7.6 Hz), 130.66, 129.79, 127.96 (d, *J*_C–F_ = 9.1 Hz), 122.58 (d, *J*_C–F_ = 24.2 Hz), 107.95 (d, *J*_C–F_ = 27.2 Hz), 23.25, 18.47; MS (ESI) *m/z* calcd. for C_16_H_16_FN_2_O_2_S [M + H]^+^ 319.1, found 319.0.

*3-Bromomethyl-2-(2,6-dimethylphenyl)-2H-benzo[e][1,2,4]thiadiazine1,1-dioxide* (**11**) 

Compound **9** (1.0 g, 3.3 mmol) was dissolved in glacial acetic acid (10 mL), and then NBS (0.3 g, 1.65 mmol) was added. After the mixture was stirred at room temperature for 0.5 h, distilled water (50 mL) was added. The mixture was extracted by dichloromethane, washed with brine, dried over anhydrous Na_2_SO_4_. After filtration and evaporation, the residue was purified by silica gel chromatography (EtOAc/hexane = 1:10) to give **11** (0.5 g) with a conversion yield of 79% as a white solid, m.p. 150–151°C; ^1^H-NMR (CDCl_3_) δ 7.90 (dd, *J* = 7.8, 1.2 Hz, 1H, Ar-H), 7.75 (td, *J* = 8.4, 1.2 Hz, 1H, Ar-H), 7.69 (dd, *J* = 7.8, 0.6 Hz, 1H, Ar-H), 7.55 (td, *J* = 8.4, 1.2 Hz, 1H, Ar-H), 7.29 (t, *J* = 7.8 Hz, 1H, Ar-H), 7.19 (d, *J* = 7.8 Hz, 2H, Ar-H), 3.97 (s, 2H, CH_2_Br), 2.24 (s, 6H, 2,6-(CH_3_)_2_); ^13^C-NMR (CDCl_3_) δ 151.86, 142.16, 138.89, 133.69, 132.14, 130.36, 129.51, 128.37, 128.25, 127.79, 121.00, 28.91, 18.95; MS (ESI) *m/z* calcd. for C_16_H_16_BrN_2_O_2_S [M + H]^+^ 379.0 and 381.0, found 381.3 and 383.4.

*3-Bromomethyl-2-(2,6-dimethylphenyl)-7-fluoro-2H-benzo[e][1,2,4]thiadiazine 1,1-dioxide* (**12**) 

According to the procedures described for the synthesis of **11**, compound **12** were obtained as a colorless solid (0.65 g) in 72% conversion yield, m.p. 185–186 °C; ^1^H-NMR (DMSO-d_6_) δ 7.95 (dd, *J* = 8.4, 3.0 Hz, 1H, Ar-H), 7.84 (dd, *J* = 10.8, 5.4 Hz, 1H, Ar-H), 7.79 (td, *J* = 10.8, 3.0 Hz, 1H, Ar-H), 7.37 (t, *J* = 8.4 Hz, 1H, Ar-H), 7.29 (d, *J* = 9.0 Hz, 2H, Ar-H), 4.10 (s, 2H, CH_2_Br), 2.14 (s, 6H, 2,6-(CH_3_)_2_); ^13^C-NMR (DMSO-d_6_) δ 161.21 (d, *J*_C–F_ = 250.6 Hz), 151.77, 138.82, 132.20, 131.54 (d, *J*_C–F_ = 9.1 Hz), 130.94, 129.97, 129.28, 128.60 (d, *J*_C–F_ = 9.1 Hz), 122.87 (d, *J*_C–F_ = 24.2 Hz), 108.32 (d, *J*_C–F_ = 25.7 Hz), 30.02, 18.72; MS (ESI) *m/z* calcd.for C_16_H_15_BrFN_2_O_2_S [M + H]^+^ 397.0 and 399.0, found 397.2 and 399.1.

*3-((4-Amino-3-iodo-1H-pyrazolo[3,4-d]pyrimidin-1-yl)methyl)-2-(2,6-dimethylphenyl)-2H-benzo[e]-[1,2,4]thiadiazine 1,1-dioxide* (**13**) 

To a solution of **11** (1.1 g, 2.9 mmol) and 3-iodo-1*H*-pyrazolo[3,4-d]pyrimidin-4-amine (1.1 g, 4.4 mmol) in DMF (8 mL), K_2_CO_3_ (0.8 g, 5.8 mmol) was added. After stirring at 60 °C for 5 h, the mixture was poured into water (100 mL), extracted by EtOAc. The organic layer was washed with brine, dried over anhydrous Na_2_SO_4_. After filtration and evaporation, the residue was purified by silica gel chromatography (EtOAc/hexane = 1:10) to give **13** (1.4 g, 86%) as a white solid, m.p. 242–243 °C; ^1^H-NMR (DMSO-d_6_) δ 8.07 (s, 1H, Ar-H), 7.94 (dd, *J* = 7.8, 1.2 Hz, 1H, Ar-H), 7.80 (td, *J* = 7.8, 1.2 Hz, 1H, Ar-H), 7.65 (td, *J* = 7.8, 1.2 Hz, 1H, Ar-H), 7.43 (d, *J* = 7.8 Hz, 1H, Ar-H), 7.25 (t, *J* = 7.2 Hz, 1H, Ar-H), 7.17 (d, *J*= 7.2 Hz, 2H, Ar-H), 5.12 (s, 2H, NCH_2_), 2.07 (s, 6H, 2,6-(CH_3_)_2_); ^13^C-NMR (CDCl_3_) δ 162.77, 161.35, 159.35, 156.17, 146.35, 143.45, 139.66, 136.44, 135.32, 134.48, 133.91, 133.26, 132.44, 108.30, 95.82, 60.13, 54.33, 23.17; MS (ESI) *m/z* calcd. for C_21_H_19_IN_7_O_2_S [M + H]^+^ 560.0, found 560.2.

*3-((4-Amino-3-iodo-1H-pyrazolo[3,4-d]pyrimidin-1-yl)methyl)-2-(2,6-dimethylphenyl)-7-fluoro-2H-benzo-[e][1,2,4]thiadiazine 1,1-dioxide* (**14**) 

Following the procedures described for the synthesis of **13**, compound **14** were obtained as a white solid (1.6 g) in 58% yield, m.p. 243–244 °C; ^1^H-NMR (DMSO-d_6_) δ 8.08 (s, 1H, Ar-H), 7.92 (dd, *J* = 11.4, 4.2 Hz, 1H, Ar-H), 7.68 (td, *J* = 13.2, 4.2 Hz, 1H, Ar-H), 7.54 (dd, *J* = 13.2, 7.2 Hz, 1H, Ar-H), 7.26 (dd, *J* = 13.2, 10.2 Hz, 1H, Ar-H), 7.17 (d, *J* = 11.4 Hz, 2H, Ar-H), 5.13 (s, 2H, NCH_2_), 2.06 (s, 6H, 2,6-(CH_3_)_2_); ^13^C-NMR (DMSO-d_6_) δ 160.89 (d, *J*_C–F_ = 250.7 Hz), 158.02, 156.61, 154.57, 150.89, 138.69, 131.55, 131.46 (d, *J*_C–F_ = 7.6 Hz), 130.64, 129.76, 128.54 (d, *J*_C–F_ = 7.6 Hz), 122.71 (d, *J*_C–F_ = 22.7 Hz), 108.28 (d, *J*_C–F_ = 25.7 Hz), 103.55, 91.13, 79.59, 49.55, 18.40; MS (ESI) *m/z* calcd. for C_21_H_18_FIN_7_O_2_S [M + H]^+^ 578.0, found 578.0.

*3-((4-Amino-3-(1H-indol-5-yl)-1H-pyrazolo[3,4-d]pyrimidin-1-yl)methyl)-2-(2,6-dimethylphenyl)-2H-benzo[e][1,2,4]thiadiazine 1,1-dioxide* (**15a**) 

To a solution of **13** (180 mg, 0.30 mmol) in dioxane (4 mL) and distilled water (1.5 mL) was added 1*H*-indol-5-ylboronic acid (88 mg, 0.55 mmol), sodium carbonate anhydrous (103 mg, 0.97 mmol) and Pd(PPh_3_)_4_ (12 mg, 0.03 mmol). The mixture was degassed with N_2_, and refluxed for 4 h. After cooling to room temperature, EtOAc (50 mL) and distilled water (10 mL) were added, and the organic layer was washed brine, dried over Na_2_SO_4_. After filtration and evaporation, the residue was purified by silica gel chromatography (EtOAc/hexane = 1:1) to give **15a** (161 mg, 91%) as a white solid, m.p. 216–217 °C; ^1^H-NMR (CDCl_3_) δ 11.32 (s, 1H, NH), 8.09 (s, 1H, Ar-H), 7.95 (dd, *J* = 7.8, 1.2 Hz, 1H, Ar-H), 7.81 (td, *J* = 7.8, 1.2 Hz, 2H, Ar-H), 7.65 (td, *J* = 7.8, 0.6 Hz, 1H, Ar-H), 7.57 (d, *J* = 8.4 Hz, 1H, Ar-H), 7.52 (d, *J* = 7.8 Hz, 1H, Ar-H), 7.45 (t, *J* = 3.0 Hz, 1H, Ar-H), 7.38 (dd, *J* = 8.4, 1.8 Hz, 1H, Ar-H), 7.25 (t, *J* = 7.8 Hz, 1H, Ar-H), 7.15 (d, *J* = 7.8 Hz, 2H, Ar-H), 6.55 (t, *J* = 8.4, 1.8 Hz, 1H, Ar-H), 5.20 (s, 2H, NCH_2_), 2.04 (s, 6H, 2,6-(CH_3_)_2_); ^13^C-NMR (CDCl3) δ 158.53, 156.22, 155.43, 152.04, 146.79, 141.79, 138.70, 136.53, 134.86, 131.96, 130.48, 129.70, 129.09, 128.51, 128.47, 127.83, 127.04, 123.90, 121.76, 121.20, 120.52, 112.68, 102.22, 97.86, 49.61, 18.35; HRMS (ESI) *m/z* calcd. for C_29_H_25_N_8_O_2_S [M + H]^+^ 549.1816, found 549.1829.

*3-((4-Amino-3-(3,4-dimethoxyphenyl)-1H-pyrazolo[3,4-d]pyrimidin-1-yl)methyl)-2-(2,6-dimethylphenyl)-2H-benzo[e][1,2,4]thiadiazine 1,1-dioxide* (**15b**) 

According to the procedures described for the synthesis of **15a**, compound **15b** were obtained as a white solid (61 mg) in 75% yield, m.p. 220–222 °C; ^1^H-NMR (DMSO-d_6_) δ 8.31 (s, 1H, NH), 8.09 (s, 1H, Ar-H), 7.94 (dd, *J* = 7.8, 1.2 Hz, 1H, Ar-H), 7.81 (td, *J* = 7.8, 1.2 Hz, 1H, Ar-H), 7.65 (td, *J* = 7.2, 1.2 Hz, 1H, Ar-H), 7.50 (d, *J* = 8.4 Hz, 1H, Ar-H), 7.26 (t, *J* = 7.8 Hz, 1H, Ar-H), 7.15–7.19 (m, 4H, Ar-H), 7.12 (d, *J* = 8.4 Hz, 1H, Ar-H), 5.18 (s, 2H, NCH_2_), 3.81 (s, 3H, OCH_3_), 3.80 (s, 3H, OCH_3_), 2.02 (s, 6H, 2,6-(CH_3_)_2_); ^13^C-NMR (DMSO-d_6_) δ 158.50, 156.26, 155.50, 151.98, 149.83, 149.51, 145.20, 141.74, 138.71, 134.86, 131.98, 130.50, 129.70, 129.13, 128.47, 127.84, 125.53, 121.19, 121.03, 112.71, 111.99, 97.70, 79.63, 56.04, 55.89, 49.64, 18.32; HRMS (ESI) *m/z* calcd. for C_29_H_28_N_7_O_4_S [M + H]^+^ 570.1918, found 570.1921.

*3-((4-Amino-3-(3-fluoro-4-methoxyphenyl)-1H-pyrazolo[3,4-d]pyrimidin-1-yl)methyl)-2-(2,6-dimethyl-phenyl)-2H-benzo[e][1,2,4]thiadiazine 1,1-dioxide* (**15c**) 

According to the procedures described for the synthesis of **15a**, compound **15c** were obtained as a white solid (59 mg) in 66% yield, m.p. 226–227 °C; ^1^H-NMR (DMSO-d_6_) δ 8.31 (s, 1H, NH), 8.10 (s, 1H, Ar-H), 7.94 (d, *J* = 7.8 Hz, 1H, Ar-H), 7.81 (t, *J* = 7.8 Hz, 1H, Ar-H), 7.65 (t, *J* = 7.8 Hz, 1H, Ar-H), 7.49 (d, *J* = 8.4 Hz, 1H, Ar-H), 7.43–7.38 (m, 2H, Ar-H), 7.33 (t, *J* = 8.4 Hz, 1H, Ar-H), 7.25 (t, *J* = 7.8 Hz, 1H, Ar-H), 7.15 (d, *J* = 7.8 Hz, 2H, Ar-H), 5.18 (s, 2H, NCH_2_), 3.90 (s, 3H, OCH_3_), 2.02 (s, 6H, 2,6-(CH_3_)_2_); ^13^C-NMR (DMSO-d_6_) δ 158.50, 156.33, 155.58, 152.84, 152.03 (d, *J*_C–F_ = 244.6 Hz), 151.85, 148.13 (d, *J*_C–F_ = 9.1 Hz), 144.02, 141.72, 138.69, 134.86, 131.93, 130.51, 129.71, 128.82 (d, *J*_C–F_ = 95.1 Hz), 127.82, 125.78 (d, *J*_C–F_ = 6.0 Hz), 125.21, 121.20, 116.11 (d, *J*_C–F_ = 19.6 Hz), 114.91, 97.67, 79.65, 56.57, 49.64, 18.32; HRMS (ESI) *m/z* calcd. for C_28_H_25_FN_7_O_3_S [M + H]^+^ 558.1718, found 558.1733.

*3-((4-Amino-3-(6-methoxypyridin-3-yl)-1H-pyrazolo[3,4-d]pyrimidin-1-yl)methyl)-2-(2,6-dimethylphenyl)-2H-benzo[e][1,2,4]thiadiazine 1,1-dioxide* (**15d**) 

According to the procedures described for the synthesis of **15a**, compound **15d** were obtained as a white solid (41 mg) in 47% yield, m.p. 248–249 °C; ^1^H-NMR (DMSO-d_6_) δ 8.39 (d, *J* = 1.8 Hz, 1H, Ar-H), 8.31 (s, 1H, NH), 8.11 (s, 1H, Ar-H), 7.94 (dd, *J* = 7.8, 1.2 Hz, 1H, Ar-H), 7.92 (dd, *J* = 8.4, 2.4 Hz, 1H, Ar-H), 7.81 (td, *J* = 8.4, 1.2 Hz, 1H, Ar-H), 7.65 (td, *J* =7.8, 1.2 Hz, 1H, Ar-H), 7.49 (d, *J* = 7.8 Hz, 1H, Ar-H), 7.26 (t, *J* = 7.8 Hz, 1H, Ar-H), 7.16 (d, *J* = 7.8 Hz, 2H, Ar-H), 6.98 (d, *J* = 8.4 Hz, 1H, Ar-H), 5.20 (s, 2H, NCH_2_), 3.92 (s, 3H, OCH_3_), 2.03 (s, 6H, 2,6-(CH_3_)_2_); ^13^C-NMR (DMSO-d_6_) δ 164.14, 158.60, 156.38, 155.61, 151.81, 146.69, 142.36, 141.71, 139.29, 138.69, 134.87, 131.91, 130.51, 129.70, 129.13, 128.50, 127.80, 122.70, 121.19, 111.48, 97.92, 79.64, 53.89, 49.66, 18.33; HRMS (ESI) *m/z* calcd. for C_27_H_25_N_8_O_4_S [M + H]^+^ 541.1765, found 541.1781.

*3-((4-Amino-3-(2,3-dihydrobenzo[b][1,4]dioxin-6-yl)-1H-pyrazolo[3,4-d]pyrimidin-1-yl)methyl)-2-(2,6-dimethylphenyl)-2H-benzo[e][1,2,4]thiadiazine 1,1-dioxide* (**15e**) 

According to the procedures described for the synthesis of **15a**, compound **15e** were obtained as a white solid (71 mg) in 64% yield, m.p. 225–226 °C; ^1^H-NMR (DMSO-d_6_) δ 8.31 (s, 1H, NH), 8.08 (s, 1H, Ar-H), 7.94 (dd, *J* = 7.8, 1.2 Hz, 1H, Ar-H), 7.82 (td, *J* = 7.8, 1.2 Hz, 1H, Ar-H), 7.65 (td, *J* = 7.8, 1.2 Hz, 1H, Ar-H), 7.51 (d, *J* = 8.4 Hz, 1H, Ar-H), 7.24 (t, *J* = 8.4 Hz, 1H, Ar-H), 7.14 (d, *J* = 7.8 Hz, 2H, Ar-H), 7.11–7.09 (m, 2H, Ar-H), 7.02 (d, *J* = 7.8 Hz, 1H, Ar-H), 5.18 (s, 2H, NCH_2_), 4.30 (s, 4H, OCH_2_CH_2_O), 2.01 (s, 6H, 2,6-(CH_3_)_2_); ^13^C-NMR (DMSO-d_6_) δ 158.45, 156.25, 155.47, 151.92, 144.79, 144.60, 144.23, 141.74, 138.66, 134.86, 131.94, 130.47, 129.68, 129.12, 128.48, 127.83, 126.18, 121.59, 121.19, 118.30, 117.23, 97.63, 79.64, 64.67, 64.59, 49.62, 18.30; HRMS (ESI) *m/z* calcd. for C_29_H_26_N_7_O_4_S [M + H]^+^ 568.1761, found 568.1775.

*3-((4-Amino-3-(benzo[d][1,3]dioxol-5-yl)-1H-pyrazolo[3,4-d]pyrimidin-1-yl)methyl)-2-(2,6-dimethyl-phenyl)-2H-benzo[e][1,2,4]thiadiazine 1,1-dioxide* (**15f**) 

According to the procedures described for the synthesis of **15a**, compound **15f** were obtained as a white solid (29 mg) in 34% yield, m.p. > 250 °C; ^1^H-NMR (DMSO-d_6_) δ 8.31 (s, 1H, NH), 8.08 (s, 1H, Ar-H), 7.94 (dd, *J* = 7.8, 1.2 Hz, 1H, Ar-H), 7.82 (td, *J* = 7.8, 1.2 Hz, 1H, Ar-H), 7.65 (td, *J* = 7.8, 1.2 Hz, 1H, Ar-H), 7.50 (d, *J* = 7.8 Hz, 1H, Ar-H), 7.25 (t, *J* = 7.8 Hz, 1H, Ar-H), 7.15 (d, *J* = 7.2 Hz, 2H, Ar-H), 7.12–7.09 (m, 2H, Ar-H), 7.07 (d, *J* = 8.4, 1H, Ar-H), 6.10 (s, 2H, OCH_2_O), 5.17 (s, 2H, NCH_2_), 2.01 (s, 6H, 2,6-(CH_3_)_2_); ^13^C-NMR (DMSO-d_6_) δ 158.44, 156.28, 155.47, 151.89, 148.36, 144.98, 141.73, 138.67, 134.87, 131.93, 130.49, 129.70, 129.14, 128.50, 127.82, 126.85, 122.65, 121.19, 109.44, 108.80, 101.89, 97.65, 79.64, 49.62, 18.30; HRMS (ESI) *m/z* calcd. for C_28_H_24_N_7_O_4_S [M + H]^+^ 554.1605, found 554.1614.

*3-((4-Amino-3-(4-methoxyphenyl)-1H-pyrazolo[3,4-d]pyrimidin-1-yl)methyl)-2-(2,6-dimethylphenyl)-2H-benzo[e][1,2,4]thiadiazine 1,1-dioxide* (**15g**) 

According to the procedures described for the synthesis of **15a**, compound **15g** were obtained as a white solid (45 mg) in 74% yield, m.p. 248–249 °C; ^1^H-NMR (DMSO-d_6_) δ 8.32 (s, 1H, NH), 8.09 (s, 1H, Ar-H), 7.94 (dd, *J* = 7.8, 1.2 Hz, 1H, Ar-H), 7.82 (td, *J* = 7.8, 1.2 Hz, 1H, Ar-H), 7.65 (td, *J* = 7.8, 1.2 Hz, 1H, Ar-H), 7.57 (d, *J* = 8.4 Hz, 2H, Ar-H), 7.50 (d, *J* = 8.4 Hz, 1H, Ar-H), 7.25 (t, *J* = 7.8 Hz, 1H, Ar-H), 7.15 (d, *J* = 7.8 Hz, 2H, Ar-H), 7.11 (d, *J* = 8.4 Hz, 2H, Ar-H), 5.19 (s, 2H, NCH_2_), 3.82 (s, 3H, OCH_3_), 2.02 (s, 6H, 2,6-(CH_3_)_2_); ^13^C-NMR (DMSO-d_6_) δ160.18, 158.51, 156.26, 155.51, 151.92, 145.03, 141.75, 138.69, 134.85, 131.93, 130.48, 129.95, 129.69, 129.10, 128.49, 127.82, 125.43, 121.19, 115.12, 97.70, 79.63, 55.72, 49.60, 18.34; HRMS (ESI) *m/z* calcd. for C_28_H_26_N_7_O_3_S [M + H]^+^ 540.1812, found 540.1829.

*3-((4-Amino-3-(3-methoxyphenyl)-1H-pyrazolo[3,4-d]pyrimidin-1-yl)methyl)-2-(2,6-dimethylphenyl)-2H-benzo[e][1,2,4]thiadiazine 1,1-dioxide* (**15h**) 

According to the procedures described for the synthesis of **15a**, compound **15h** were obtained as a white solid (46 mg) in 59% yield, m.p. 221–222 °C; ^1^H-NMR (DMSO-d_6_) δ 8.31 (s, 1H), 8.10 (s, 1H, Ar-H), 7.94 (dd, *J* = 7.8, 1.2 Hz, 1H, Ar-H), 7.82 (td, *J* = 7.8, 1.2 Hz, 1H, Ar-H), 7.63 (td, *J* = 7.8, 1.2 Hz, 1H, Ar-H), 7.50 (d, *J* = 7.8 Hz, 1H, Ar-H), 7.47 (t, *J* = 7.8 Hz, 1H, Ar-H), 7.26 (t, *J* = 7.8 Hz, 1H, Ar-H), 7.23 (d, *J* = 7.8 Hz, 1H, Ar-H), 7.20–7.11 (m, 3H, Ar-H), 7.06 (dd, *J* = 8.4, 2.4 Hz, 1H, Ar-H), 5.21 (s, 2H, NCH_2_), 3.82 (s, 3H, OCH_3_), 2.02 (s, 6H, 2,6-(CH_3_)_2_); ^13^C-NMR (DMSO-d_6_) δ 160.11, 158.43, 156.31, 155.56, 151.88, 145.02, 141.72, 138.70, 134.87, 134.36, 131.95, 130.86, 130.51, 129.71, 129.14, 128.48, 127.83, 121.20, 120.76, 115.19, 113.85, 97.74, 79.64, 55.63, 49.68, 18.32; HRMS (ESI) *m/z* calcd. for C_28_H_26_N_7_O_3_S [M + H]^+^ 540.1812, found 540.1825.

*3-((4-Amino-3-(4-(trifluoromethoxy)phenyl)-1H-pyrazolo[3,4-d]pyrimidin-1-yl)methyl)-2-(2,6-dimethyl-phenyl)-2H-benzo[e][1,2,4]thiadiazine 1,1-dioxide* (**15i**) 

According to the procedures described for the synthesis of **15a**, compound **15i** were obtained as a white solid (61 mg) in 61% yield, m.p. 206–207 °C; ^1^H-NMR (DMSO-d_6_) δ 8.12 (s, 1H, Ar-H), 7.94 (dd, *J* = 8.4, 1.2 Hz, 1H, Ar-H), 7.81 (dt, *J* = 8.4, 1.2 Hz, 1H, Ar-H), 7.76 (d, *J* =8.4 Hz, 2H, Ar-H), 7.65 (dt, *J* = 8.4, 0.6 Hz, 1H, Ar-H), 7.52 (d, *J* = 7.8 Hz, 2H, Ar-H), 7.48 (d, *J* = 7.8 Hz, 1H, Ar-H), 7.26 (t, *J* = 7.8 Hz, 1H, Ar-H), 7.16 (d, *J* = 7.8 Hz, 2 H, Ar-H), 5.21 (s, 2H), 2.03 (s, 6H); ^13^C-NMR (DMSO-d_6_) δ 158.52, 156.38, 155.72, 151.77, 149.08, 143.91, 141.70, 138.70, 134.87, 132.28, 131.90, 130.59, 130.53, 129.72, 129.14, 128.50, 127.80, 122.10, 121.21, 120.57 (q, *J*_C–F_ = 256.70 Hz), 97.73, 49.68, 18.34; HRMS (ESI) *m/z* calcd. for C_28_H_23_F_3_N_7_O_3_S [M + H]^+^ 594.1530, found 594.1547.

*3-((4-Amino-3-phenyl-1H-pyrazolo[3,4-d]pyrimidin-1-yl)methyl)-2-(2,6-dimethylphenyl)-2H-benzo[e]-[1,2,4]thiadiazine 1,1-dioxide* (**15j**) 

According to the procedures described for the synthesis of **15a**, compound **15j** were obtained as a white solid (40 mg) in 73% yield, m.p. 193–194 °C; ^1^H-NMR (DMSO-d_6_) δ 8.30 (s, 1H, NH), 8.09 (s, 1H, Ar-H), 7.92 (dd, *J* = 7.8, 1.2 Hz, 1H, Ar-H), 7.80 (td, *J* = 7.8, 1.2 Hz, 1H, Ar-H), 7.66–7.62 (m, 3H, Ar-H), 7.54 (t, *J* = 7.8 Hz, 2H, Ar-H), 7.52–7.32 (m, 2H, Ar-H), 7.24 (t, *J* = 7.8 Hz, 2H, Ar-H), 7.14 (d, *J* = 7.8 Hz, 2H, Ar-H), 5.19 (s, 2H, NCH_2_), 2.01 (s, 6H, 2,6-(CH_3_)_2_); ^13^C-NMR (DMSO-d_6_) δ 1158.46, 156.31, 155.60, 151.86, 145.16, 141.72, 138.69, 134.54, 133.53, 132.51, 131.99, 131.92, 130.51, 130.47, 129.71, 129.67, 129.27, 129.20, 128.60, 127.81, 121.20, 97.73, 79.65, 49.65, 18.34; HRMS (ESI) *m/z* calcd. for C_27_H_24_N_7_O_2_S [M + H]^+^ 510.1707, found 510.1722.

*3-((4-Amino-3-(1H-indol-5-yl)-1H-pyrazolo[3,4-d]pyrimidin-1-yl)methyl)-2-(2,6-dimethylphenyl)-7-fluoro-2H-benzo[e][1,2,4]thiadiazine 1,1-dioxide* (**16a**) 

According to the procedures described for the synthesis of **15a**, compound **16a** were obtained as a white solid (65 mg) in 73% yield, m.p. 132–133 °C; ^1^H-NMR (DMSO-d_6_) δ 8.30 (s, 1H, NH), 8.08 (s, 1H, Ar-H), 7.91 (dd, *J* = 7.2, 2.4 Hz, 1H, Ar-H), 7.81 (brs, 1H, Ar-H), 7.69 (td, *J* = 9.6, 3.0 Hz, 1H, Ar-H), 7.61 (dd, *J* = 9.0, 4.8 Hz, 1H, Ar-H), 7.56 (d, *J* = 8.4 Hz, 1H, Ar-H), 7.44 (t, *J* = 3.0 Hz, 1H, Ar-H), 7.36 (dd, *J* = 8.4, 1.8 Hz, 1H, Ar-H), 7.25 (t, *J* = 7.28Hz, 1H, Ar-H), 7.16 (t, *J* = 7.8 Hz, 2H, Ar-H), 6.54 (s, 1H, 3-CH-indolyl), 5.20 (s, 2H, NCH_2_), 2.01 (s, 6H, 2,6-(CH_3_)_2_); ^13^C-NMR (DMSO-d_6_) δ 160.08 (d, *J*_C–F_ = 250.7 Hz), 158.52, 156.23, 155.41, 151.51, 146.84, 138.68, 138.61 (d, *J*_C–F_ = 3.0 Hz), 136.53, 131.81, 131.44 (d, *J*_C–F_ = 9.1 Hz), 130.55, 129.74, 128.66 (d, *J*_C–F_ = 9.1 Hz), 128.46, 127.05, 123.87, 122.67 (d, *J*_C–F_ = 24.2 Hz), 121.74, 120.52, 112.67, 108.19 (d, *J*_C–F_ = 27.2 Hz), 102.21, 97.85, 79.65, 49.57, 18.32; HRMS (ESI) *m/z* calcd. for C_29_H_24_FN_8_O_2_S [M + H]^+^ 567.1721, found 567.1739.

*3-((A-amino-3-(3,4-dimethoxyphenyl)-1H-pyrazolo[3,4-d]pyrimidin-1-yl)methyl)-2-(2,6-dimethylphenyl)-7-fluoro-2H-benzo[e][1,2,4]thiadiazine 1,1-dioxide* (**16b**) 

According to the procedures described for the synthesis of **15a**, compound **16b** were obtained as a white solid (47 mg) in 51% yield, m.p. 229–230 °C; ^1^H-NMR (DMSO-d_6_) δ 8.31 (s, 1H, NH), 8.09 (s, 1H, Ar-H), 7.91 (dd, *J* = 7.2, 2.4 Hz, 1H, Ar-H), 7.69 (td, J = 9.0, 2.4 Hz, 1H, Ar-H), 7.59 (dd, *J* = 9.0, 4.8 Hz, 1H, Ar-H), 7.26 (t, *J* = 7.8 Hz, 1H, Ar-H), 7.21–7.10 (m, 5H, Ar-H), 5.19 (s, 2H, NCH_2_), 3.82 (s, 3H, OCH_3_), 3.81 (s, 3H, OCH_3_), 2.01 (s, 6H, 2,6-(CH_3_)_2_); ^13^C-NMR (DMSO-d_6_) δ 160.78 (d, *J*_C–F_ = 250.7 Hz), 158.51, 156.27, 155.49, 151.46, 149.84, 149.52, 145.24, 138.70, 138.57 (d, *J*_C–F_ = 3.0 Hz), 131.84, 131.41 (d, *J*_C–F_ = 7.6 Hz), 130.57, 129.74, 128.69 (d, *J*_C–F_ = 7.6 Hz), 125.52, 122.66 (d, *J*_C–F_ = 24.2 Hz), 121.03, 112.72, 112.02, 108.20 (d, *J*_C–F_ = 27.2 Hz), 97.71, 79.65, 56.04, 55.89, 49.60, 18.31; HRMS (ESI) *m/z* calcd. for C_29_H_2__7_FN_7_O_4_S [M + H]^+^ 588.1824, found 588.1838.

*3-((4-Amino-3-(3-fluoro-4-methoxyphenyl)-1H-pyrazolo[3,4-d]pyrimidin-1-yl)methyl)-2-(2,6-dimethyl-phenyl)-7-fluoro-2H-benzo[e][1,2,4]thiadiazine 1,1-dioxide* (**16c**) 

According to the procedures described for the synthesis of **15a**, compound **16c** were obtained as a white solid (76 mg) in 84% yield, m.p. 201–202 °C; ^1^H-NMR (DMSO-d_6_) δ 8.31 (s, 1H, NH), 8.09 (s, 1H, Ar-H), 7.91 (dd, *J* = 7.2, 3.0 Hz, 1H, Ar-H), 7.69 (td, *J* = 7.4, 2.4 Hz, 1H, Ar-H), 7.59 (dd, *J* = 9.0, 4.2 Hz, 1H, Ar-H), 7.44–7.38 (m, 2H, Ar-H), 7.33 (t, *J* = 9.0 Hz, 1H, Ar-H), 7.26 (t, *J* = 7.2 Hz, 1H, Ar-H), 7.15 (d, *J* = 7.8 Hz, 2H, Ar-H), 5.19 (s, 2H, NCH_2_), 3.90 (s, 3H, OCH_3_), 2.01 (s, 6H, 2,6-(CH_3_)_2_); ^13^C-NMR (DMSO-d_6_) δ 160.89 (d, *J*_C–F_ = 250.7 Hz), 158.50, 156.34, 155.56, 152.02 (d, *J*_C–F_ = 246.1 Hz), 151.32, 148.14 (d, *J*_C–F_ = 10.6 Hz), 144.06, 138.68, 138.62 (d, *J*_C–F_ = 19.6 Hz), 131.78, 131.44 (d, *J*_C–F_ = 7.6 Hz), 130.59, 129.74, 128.66 (d, *J*_C–F_ = 7.6 Hz), 125.76 (d, *J*_C–F_ = 7.6 Hz), 125.21, 122.68 (d, *J*_C–F_ = 22.7 Hz), 116.11 (d, *J*_C–F_ = 19.6 Hz), 114.92, 108.21 (d, *J*_C–F_ = 25.7 Hz), 97.67, 79.63, 56.59, 49.61, 18.30; HRMS (ESI) *m/z* calcd. for C_28_H_2__4_F_2_N_7_O_3_S [M + H]^+^ 576.1624, found 576.1637.

*3-((4-Amino-3-(6-methoxypyridin-3-yl)-1H-pyrazolo[3,4-d]pyrimidin-1-yl)methyl)-2-(2,6-dimethylphenyl)-7-fluoro-2H-benzo[e][1,2,4]thiadiazine 1,1-dioxide* (**16d**) 

According to the procedures described for the synthesis of **15a**, compound **16d** were obtained as a white solid (51 mg) in 58% yield, m.p. 225–226 °C; ^1^H-NMR (DMSO-d_6_) δ 8.38 (s, 1H, NH), 8.31 (s, 1H, Ar-H), 8.10 (s, 1H, Ar-H), 7.90 (d, *J* = 8.4 Hz, 2H, Ar-H), 7.69 (t, *J* = 7.2 Hz, 1H, Ar-H), 7.59 (dd, *J* = 9.0, 4.2 Hz, 1H, Ar-H), 7.25 (t, *J* = 7.2 Hz, 1H, Ar-H), 7.15 (d, *J* = 8.4 Hz, 2H, Ar-H), 6.97 (d, *J* = 8.4 Hz, 1H, Ar-H), 5.20 (s, 2H, NCH_2_), 3.92 (s, 3H, OCH_3_), 2.01 (s, 6H, 2,6-(CH_3_)_2_); ^13^C-NMR (DMSO-d_6_) δ 164.15, 160.89 (d, *J*_C–F_ = 250.7 Hz), 158.60, 156.39, 155.59, 151.29, 146.69, 142.41, 139.29, 138.69, 138.54 (d, *J*_C–F_ = 3.0 Hz), 131.77, 131.45 (d, *J*_C–F_ = 7.6 Hz), 130.59, 129.74, 128.66 (d, *J*_C–F_ = 9.1 Hz), 122.75, 122.68, 122.67 (d, *J*_C–F_ = 22.7 Hz), 111.48, 108.20 (d, *J*_C–F_ = 27.2 Hz), 97.93, 79.64, 53.89, 49.63, 18.32; HRMS (ESI) *m/z* calcd. for C_27_H_24_FN_8_O_3_S [M + H]^+^ 559.1671, found 559.1678.

### 3.2. PI3K Kinase Assay

The ADP-Glo luminescent assay was used for PI3Kδ, PI3Kβ and PI3Kγ isoforms and the kinase-Glo luminescent assay was used for PI3Kα according to the standard protocols of Promega [40]. PI-103 was used as a positive control. The compounds were tested from 1 μM or 10 μM, 3-fold dilution, in duplicate for 10 concentrations. The kinase reaction was done in 384-well plate (Corning, Los Altos, MA, USA). Each well was loaded with test compounds (in 100% DMSO) and reaction buffer containing PI substrate. After the PI3K proteins were then added, the reaction was started by the addition of PIP2 and ATP prepared in the reaction buffer and ran for either 60 (for PI3Kα, PI3Kβ, and PI3Kγ) or 120 min (for PI3Kδ). ADP-Glo reagent was then added to terminate the reavtion. The plates were then read in a Synergy 2 reader (BioTek, Shanghai, China) for luminescence detection.

### 3.3. Cell Proliferation Assay

Cell proliferation was evaluated by The CellTiter-Glo luminescent cell viability assay (Promega, Shanghai, China) was used to evaluate the inhibitory activity of compounds **15a** and **15b** following the manufacturer’s protocol. In brief, SU-DHL-6 (ATCC) cells were seeded in 96-well plates (Corning, Los Altos, MA, USA) at density of 1 × 10^4^ cells per well, and incubated with medium alone or with the tested compounds at the indicated concentrations (50 μM in DMSO, 3-fold dilution, in duplicate for 10 concentrations). 50 μL CellTiter-Glo (Promega, Shanghai, China) reagent was added to each well to induce cell lysis, and the plate was incubated at room temperature for 10 min to stabilize luminescent signal. After 100 μL of the mixture from each well was transferred to a new 96-well black plate, the fluorescence signal was read on EnVision (Shanghai, China) and the data were analysized by XLFit 4 software (IDBS, Berlin, Germany). 

### 3.4. Molecular Docking

X-ray cocrystal structure of PI3Kδ enzyme was downloaded from RCSB Protein Date Bank (PDB ID: 2WXH) [42]. The molecular docking of **15a** and **15b** was carried out following the same procedures as reported for compounds **3** and **4** [33].

## 4. Conclusions

In a continuous study to find more potent and selective PI3Kδ inhibitors based on the rotation-limiting strategy, we substituted the carbonyl in the quinazolinone core for the sulfonyl group, designed and synthesized a series of novel 2*H*-benzo[*e*][1,2,4]thiadiazine 1,1-dioxide derivatives **15a**–**J** and **16a**–**d**. In agreement with the quinazolinone derivatives, the introduction of a 5-indolyl or 3,4-dimethoxyphenyl at the affinity pocket generated the most potent analogues **15a** and **15b** with the IC_50_ values of 217 to 266 nM, respectively. In comparison with the quinazolinone lead compounds **3** and **4**, the 2*H*-benzo[*e*][1,2,4]thiadiazine 1,1-dioxide derivatives exhibited much reduced PI3Kδ inhibitory activity, but maintained high selectivity over other PI3K isoforms. Unlike the quinazolinone lead compound **4** that was a dual PI3Kδ/γ inhibitor, the 2*H*-benzo[*e*][1,2,4]thiadiazine 1,1-dioxide **15b** was more than 21-fold selective over PI3Kγ. This may provide a structural base for the further design of more potent and selective PI3Kδ inhibitors. In agreement with their high PI3Kδ inhibitory activity, **15a** and **15b** exhibited high antiproliferative potency against B-cell leukemia SU-DHL-6 cells. 

## Figures and Tables

**Table 1:** PI3Kδ inhibitory activity of **15a**–**j** and **16a**–**d**.

Compd.	Structure	R	IC_50_ (nM) ^a^
**15a**	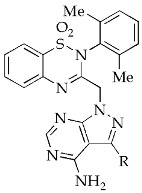	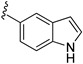	217 ± 28
**15b**		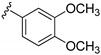	266 ± 31
**15c**		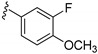	980 ± 45
**15d**		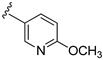	498 ± 33
**15e**		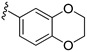	>1000
**15f**		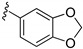	>1000
**15g**		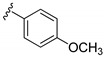	>1000
**15h**		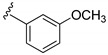	>1000
**15i**		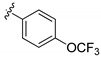	>1000
**15j**		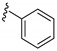	>1000
**16a**	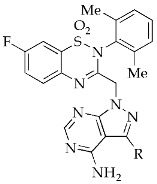	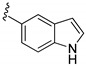	>1000
**16b**		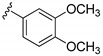	518 ± 62
**16c**		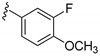	824 ± 76
**16d**		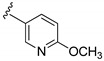	823 ± 69
PI-103			1.6 ± 0.1

^a^ The IC_50_ values are shown as the mean ± SD from two separate experiments.

**Table 2:** The isoform selectivity and SU-DHL-6 cell growth inhibitory activity of **15a** and **15b**.

Compound	IC_50_ (nM) ^a^				GI_50_ (μM) ^a^
	PI3Kα	PI3Kβ	PI3Kδ	PI3Kγ	SU-DHL-6
**15a**	>50,000	30596 ± 875	217 ± 28	>50,000	2.13 ± 0.09
**15b**	16364 ± 768	24189 ± 495	266 ± 31	5838 ± 135	2.50 ± 0.11
PI-103	6.5 ± 0.7	23 ± 1.6	1.6 ± 0.1	78 ± 4.3	0.039 ± 0.011 ^b^

^a^ The IC_50_ or GI_50_ values are shown as the mean ± SD from two separate experiments; ^b^ CAL-101 was the positive control.

## Supplementary Materials

Supplementary materials are available online.

## Abbreviations

PI3Ks	Phosphoinositide 3-kinases
PIP2	Phosphatidylinositol 4,5-bisphosphate
NBS	*N*-Bromosuccinimide
ADP	Adenosine diphosphate
ATP	Adenosine triphosphate
DMSO	Dimethyl sulfoxide
APDS	PI3Kδ syndrome
FL	Follicular lymphoma
SLL	Small lymphocytic lymphoma
CLL	Chronic lymphocytic leukemia
