# Supplementary material for: Synthesis and Evaluation of Novel 2H-Benzo[e]-[1,2,4]thiadiazine 1,1-Dioxide Derivatives as PI3Kδ Inhibitors
Source: Molecules. 2019 Nov 25;24(23):4299. doi: 10.3390/molecules24234299 (PMC6930582; doi:10.3390/molecules24234299)

Synthesis and Evaluation of Novel 2*H*-Benzo[*e*][1,2,4]thiadiazine 1,1-Dioxide Derivatives as PI3Kδ Inhibitors

Ya-Ping Gong†, Long-Qian Tang†, Tong-Shen Liu, De-Feng Wang, Zhao-Peng Liu*

Department of Medicinal Chemistry, Key Laboratory of Chemical Biology (Ministry of Education), School of Pharmaceutical Sciences, Shandong University, Jinan 250012, PR China

*Correspondence: liuzhaop@sdu.edu.cn; Tel. +86-531-88382006; Fax: +86-531-88382548

^{1}H and ^{13}C-NMR spectra and HRMS for compounds **15a**–**J** and **16a**–**d**……………………2-15

Compound 15a

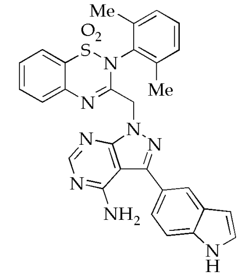

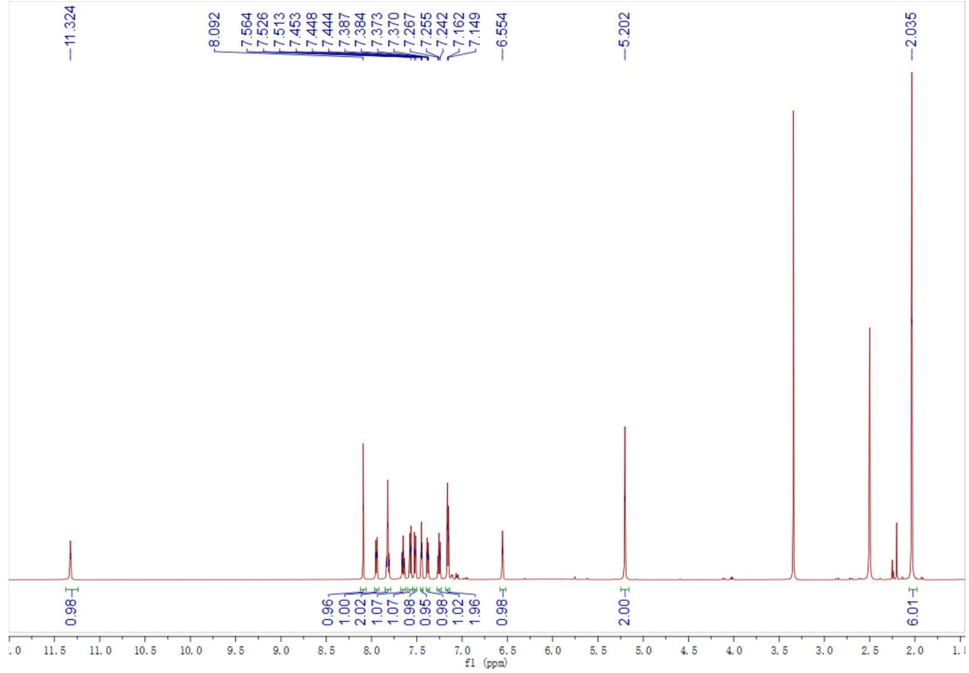

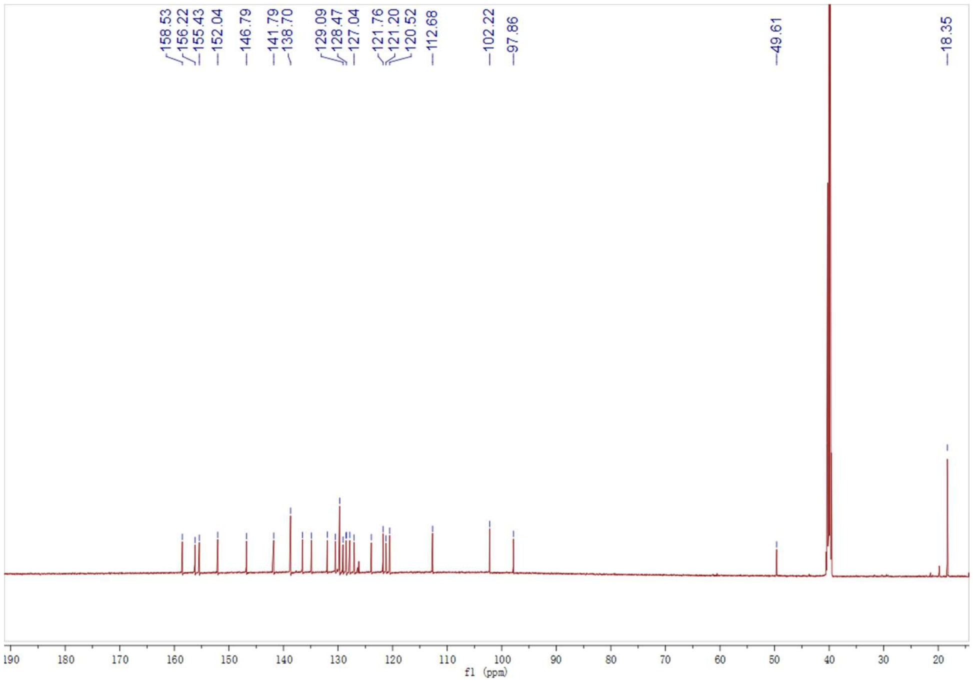

Compound 15b

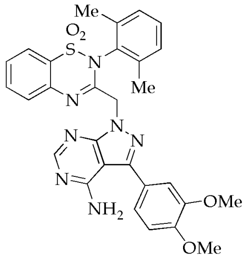

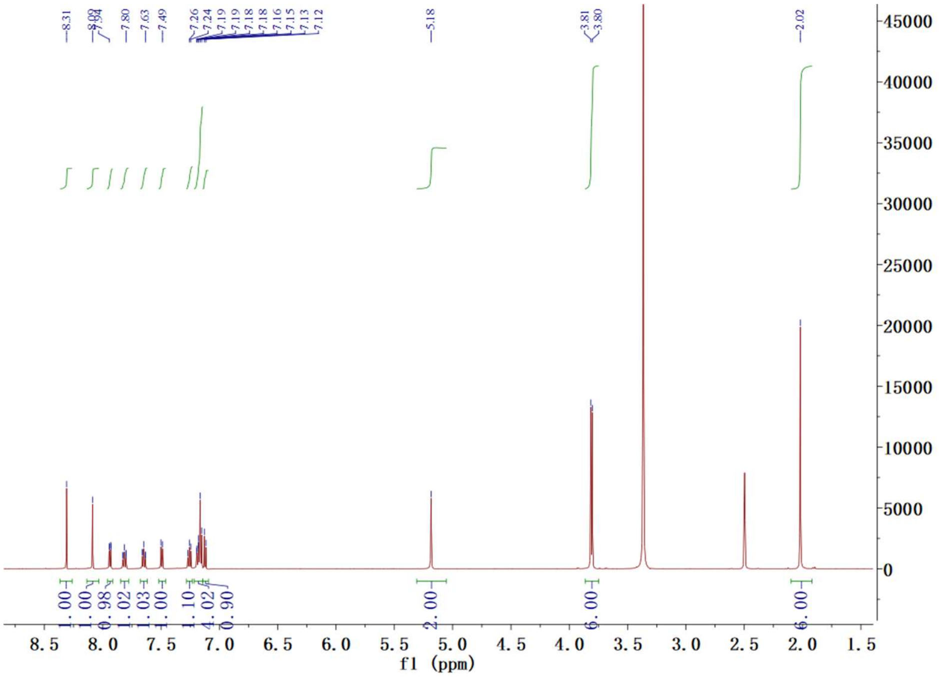

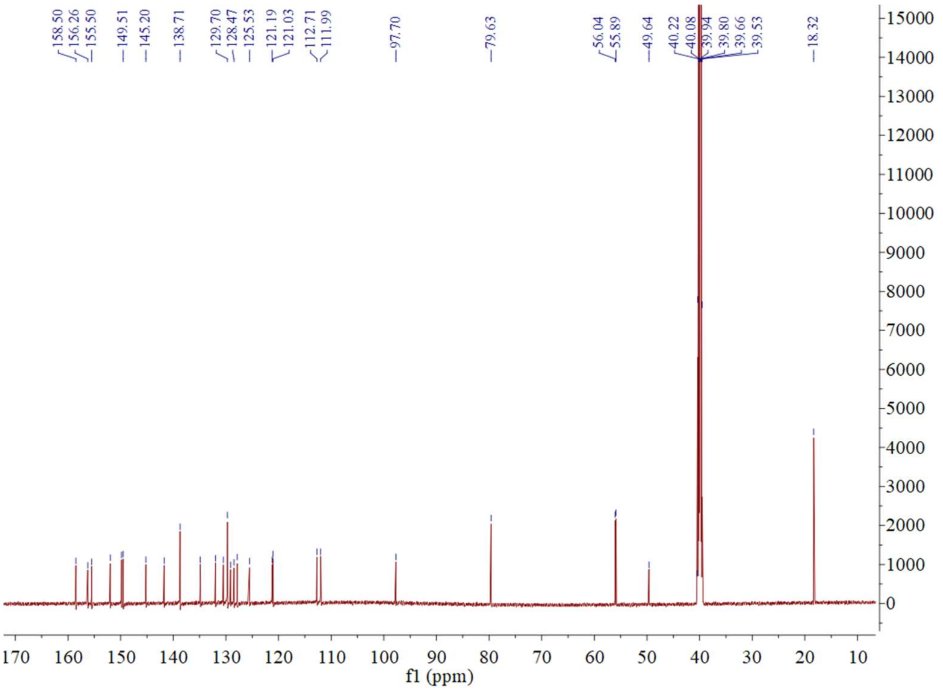

Compound 15c

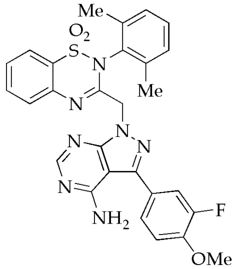

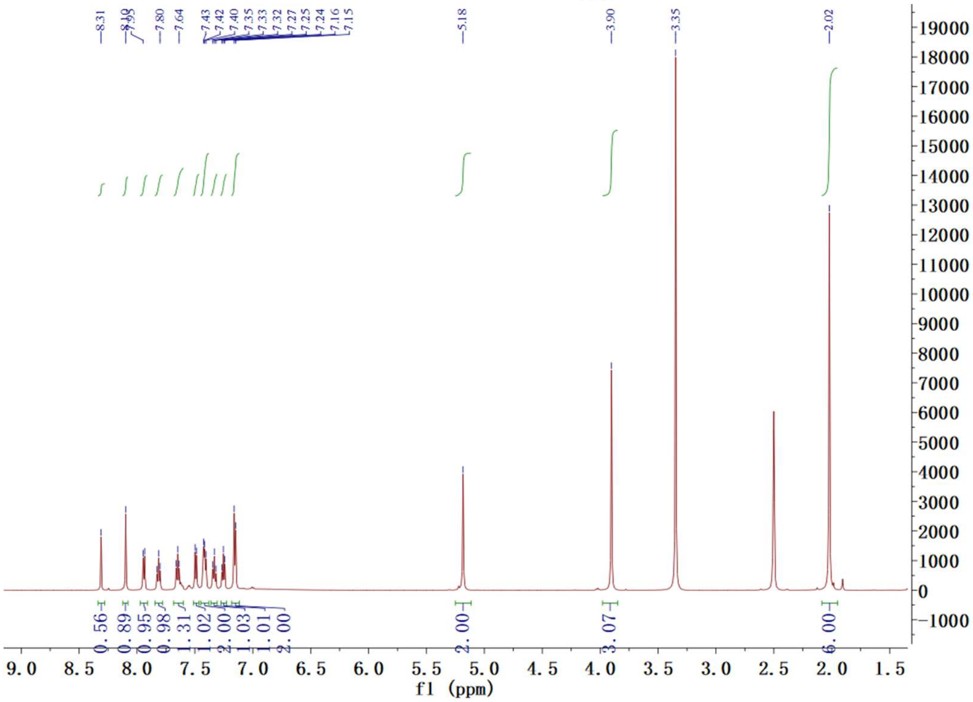

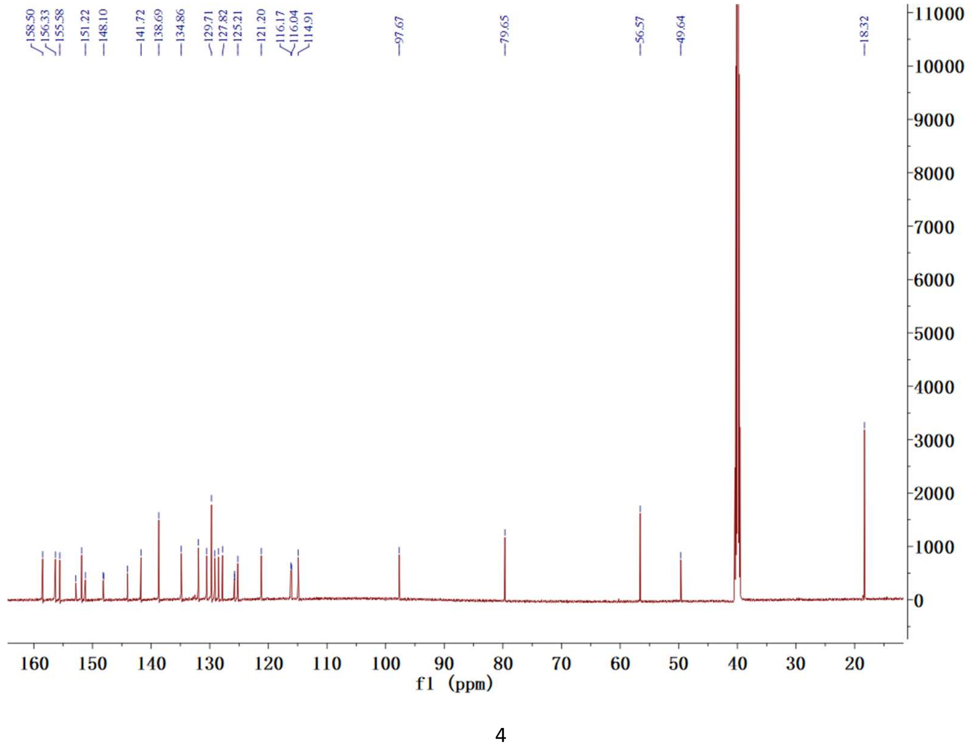

Compound 15d

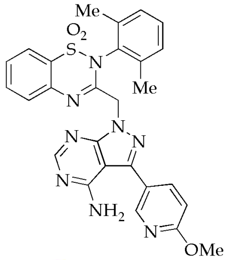

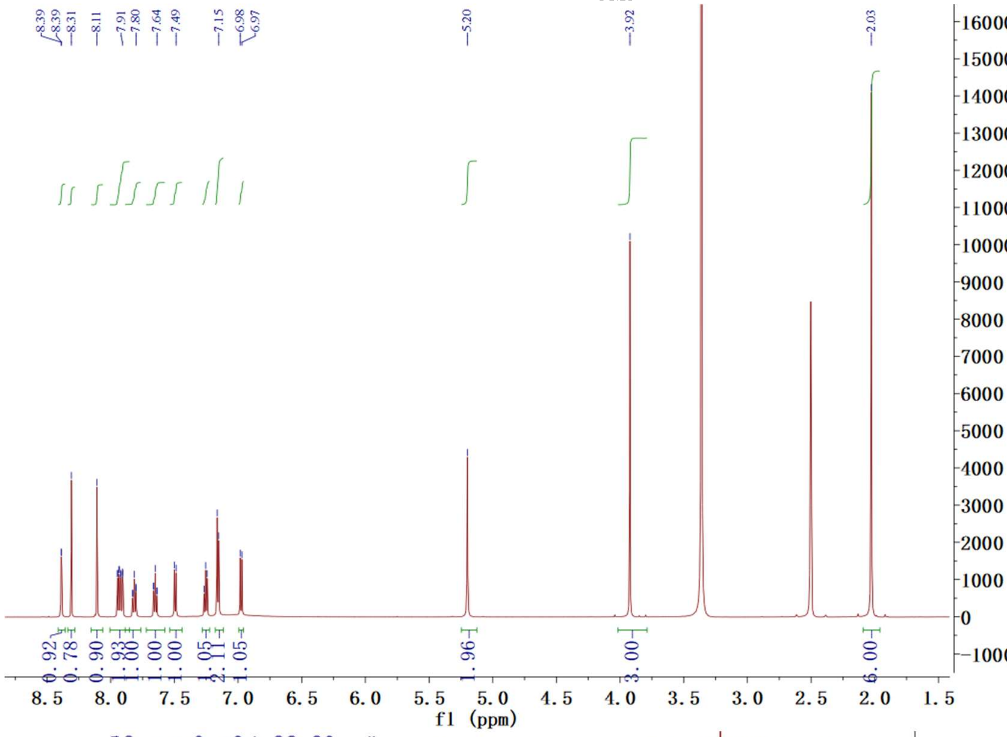

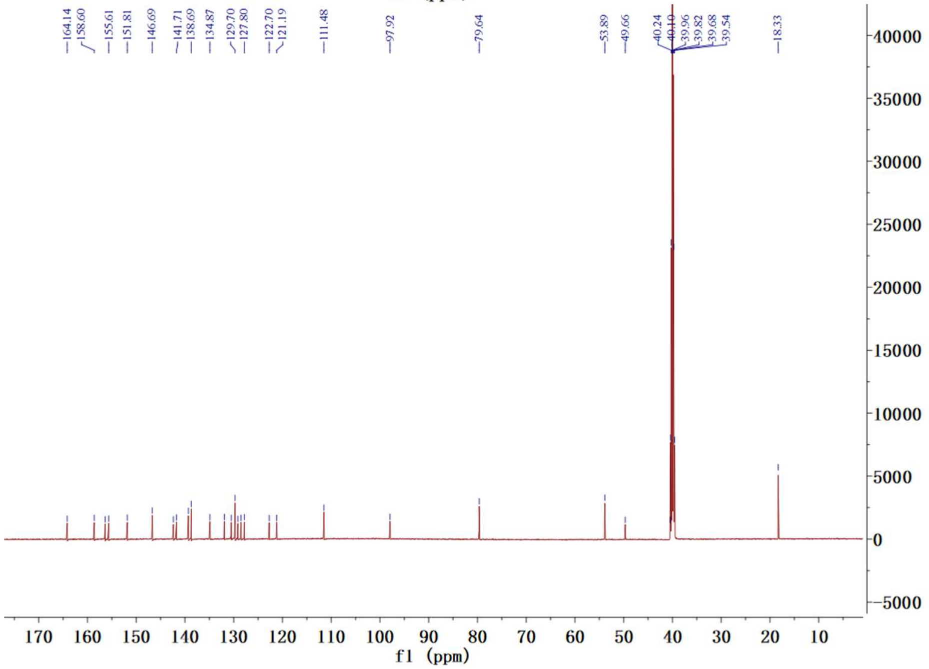

Compound 15e

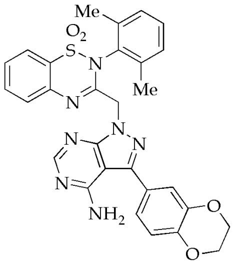

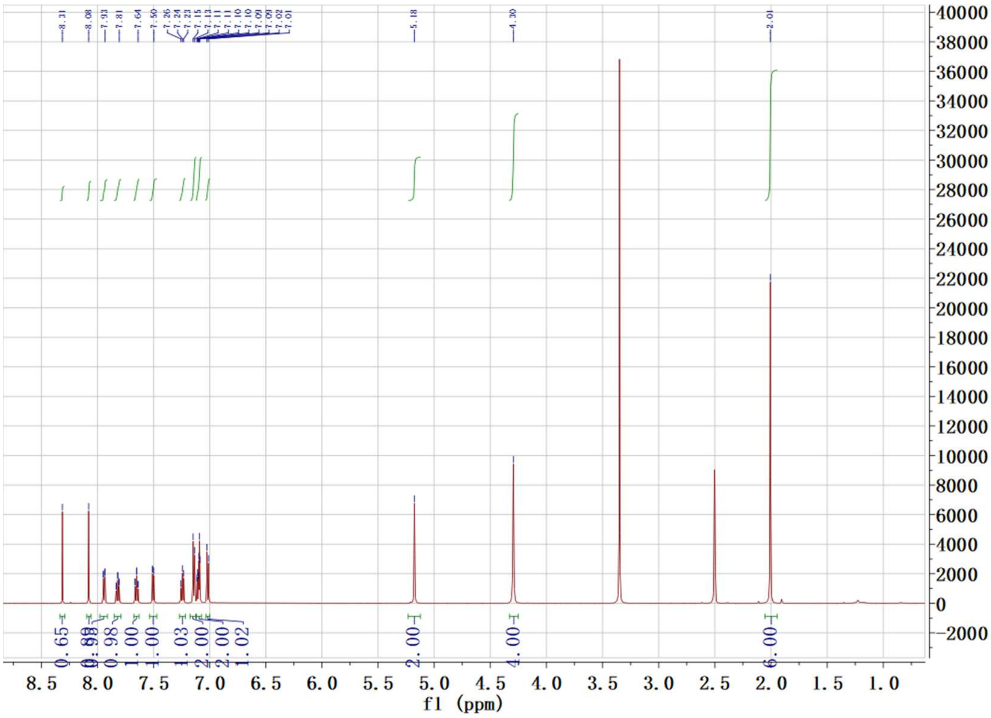

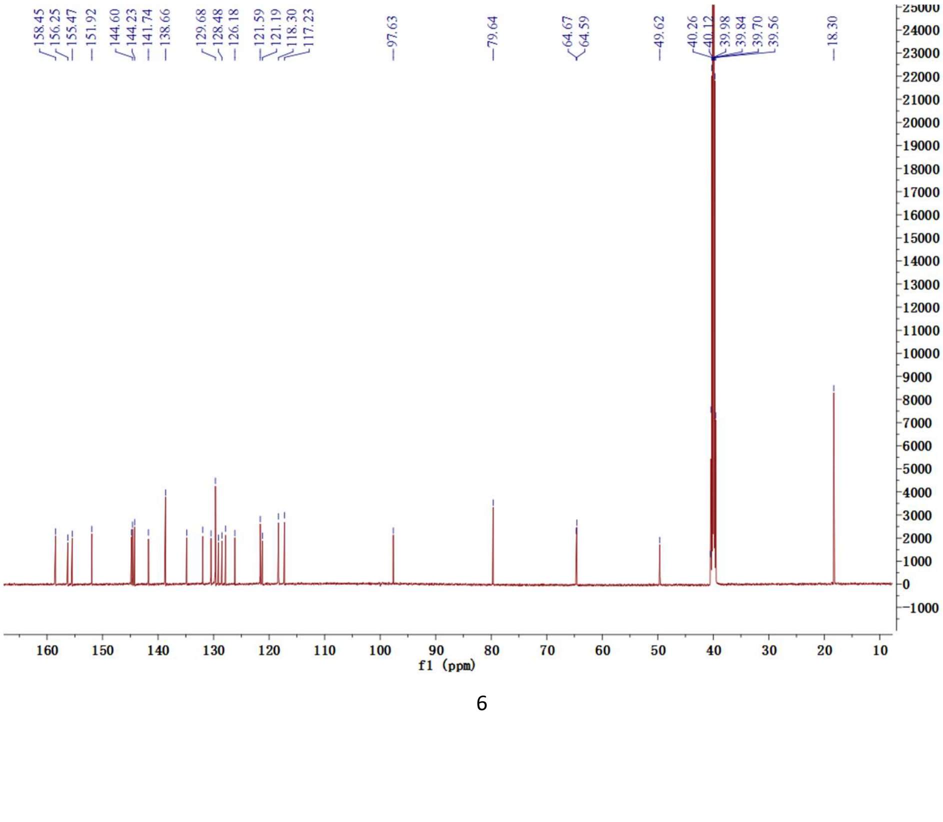

Compound 15f

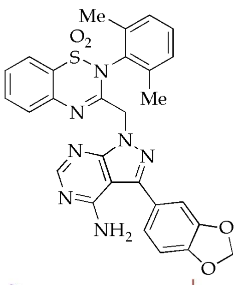

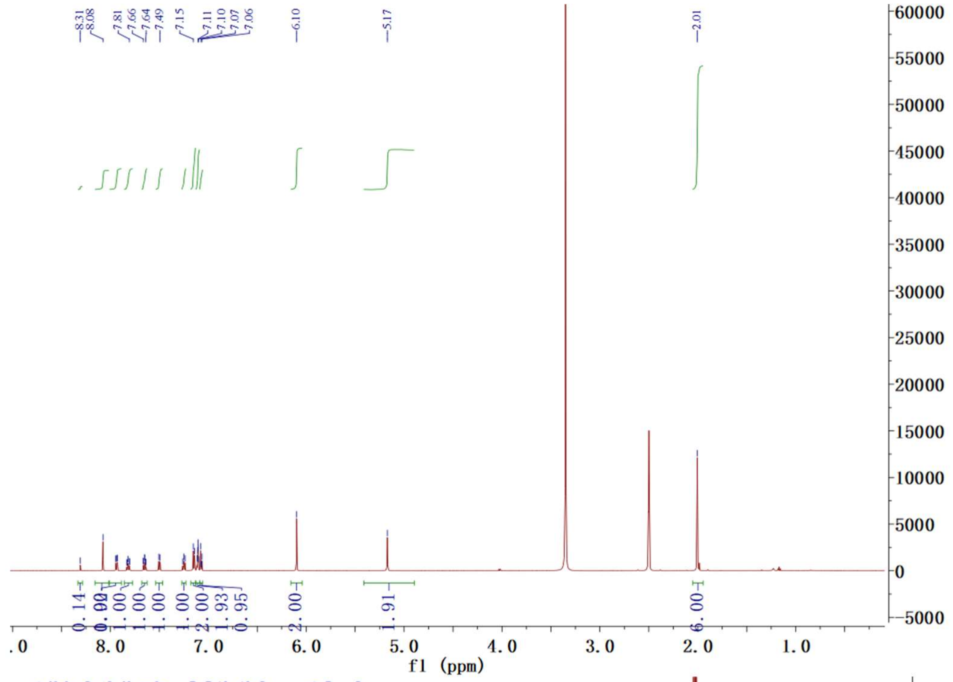

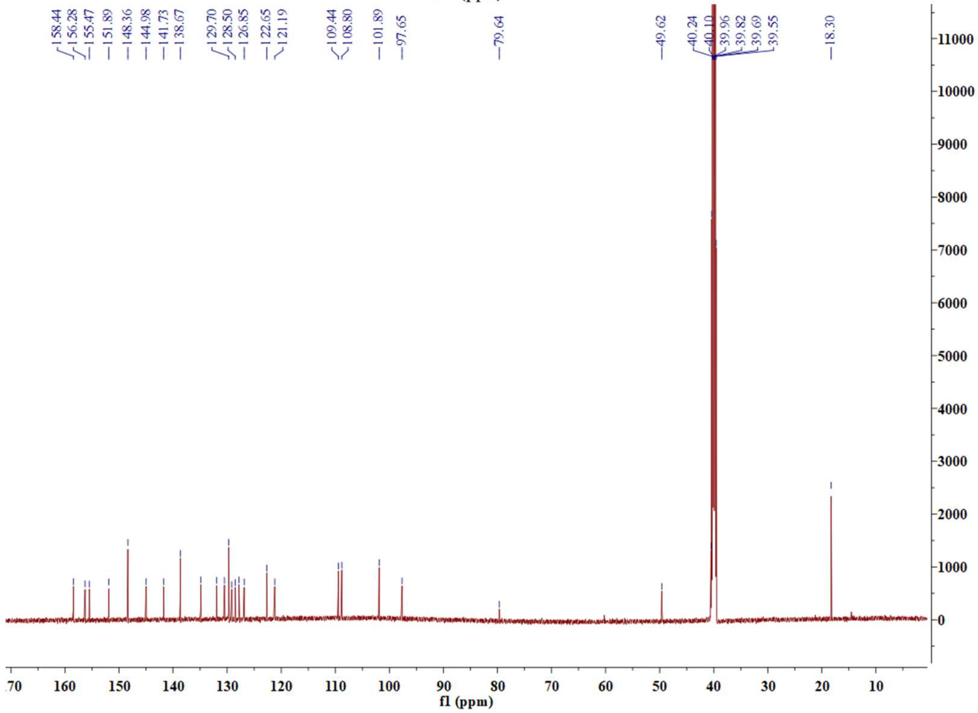

Compound 15g

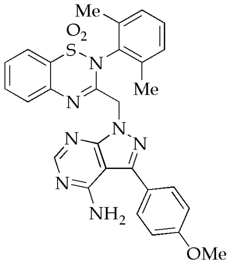

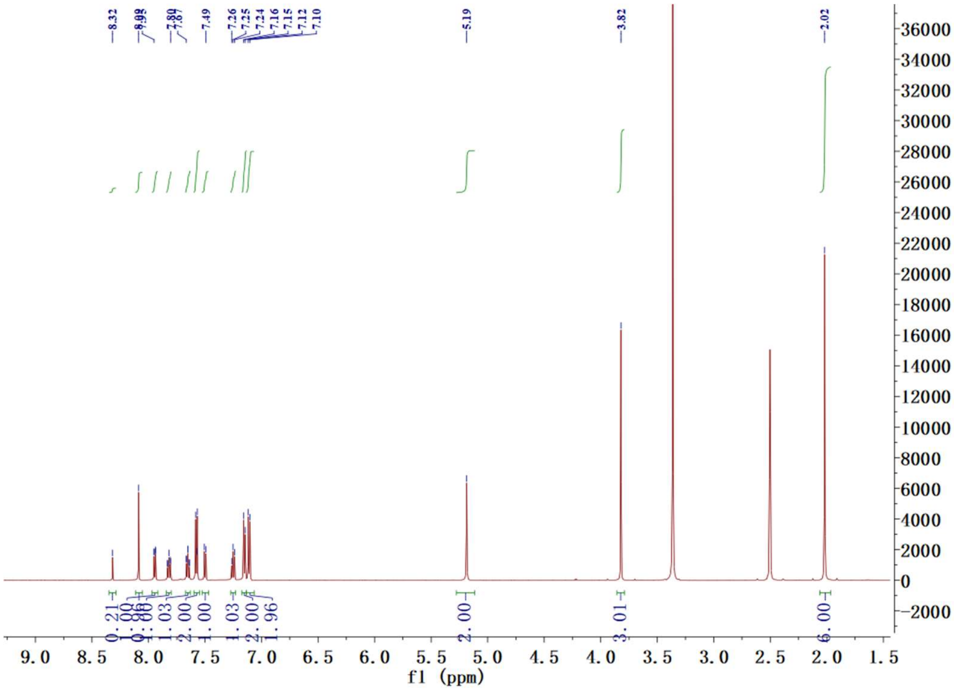

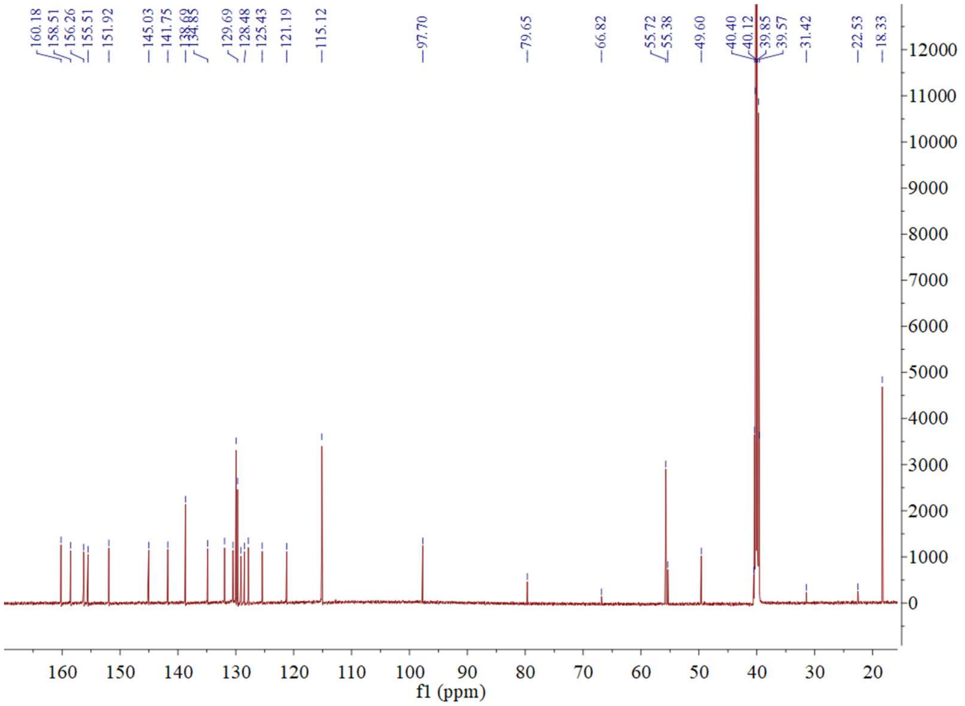

Compound 15h

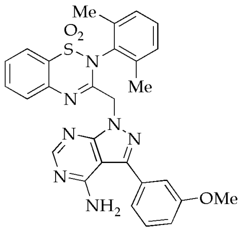

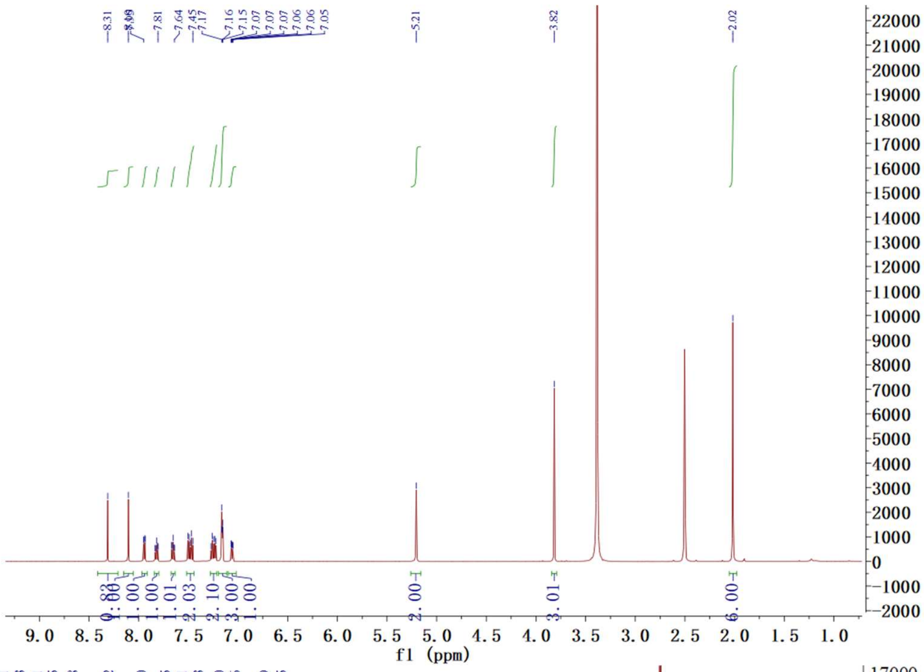

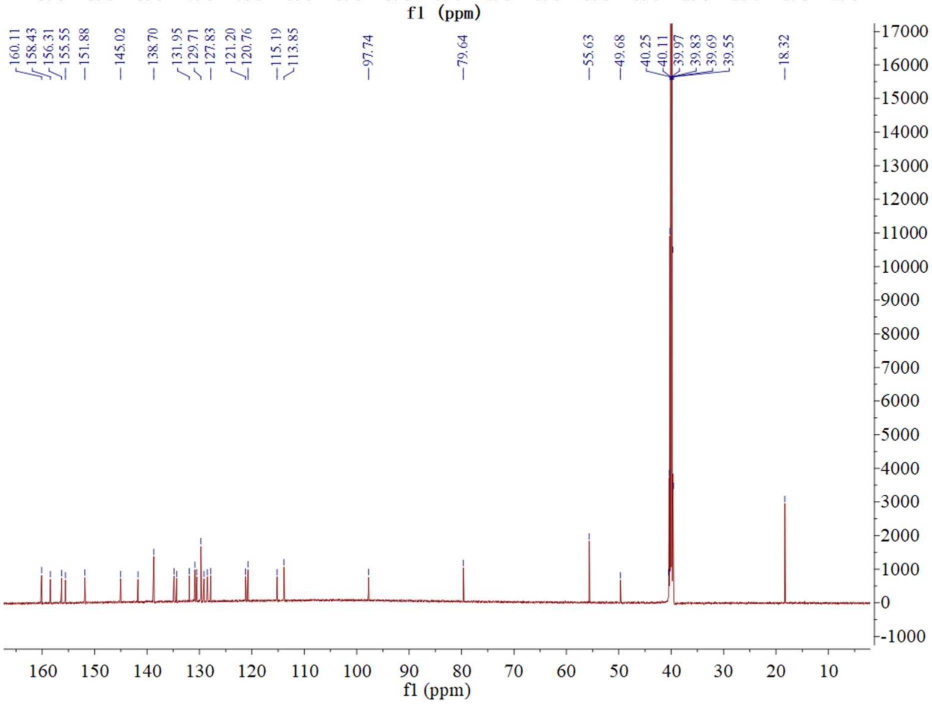

Compound 15i

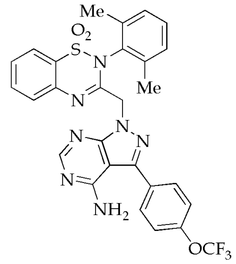

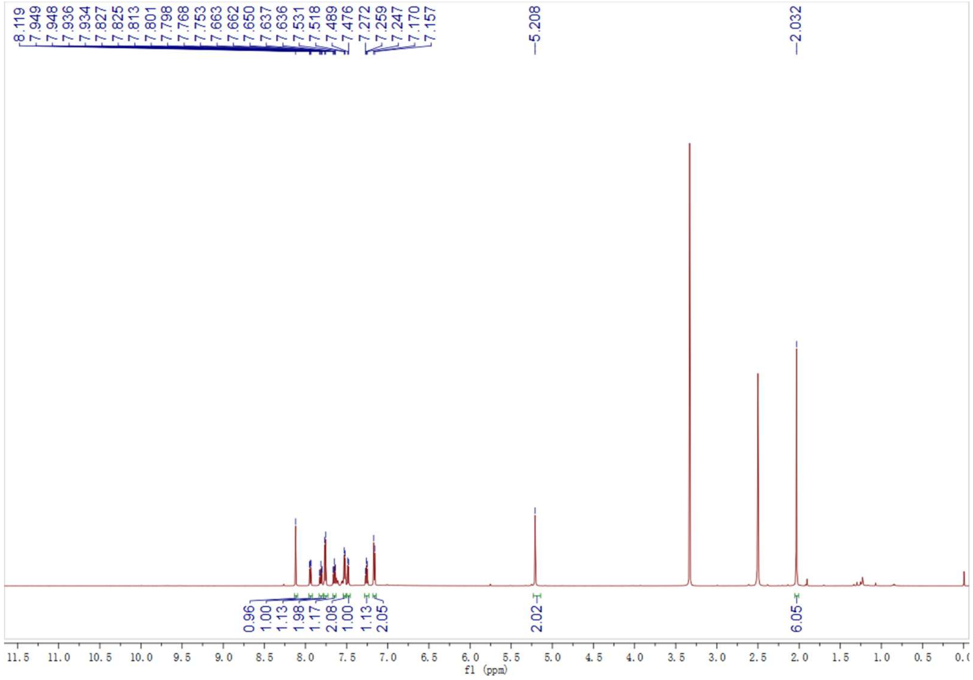

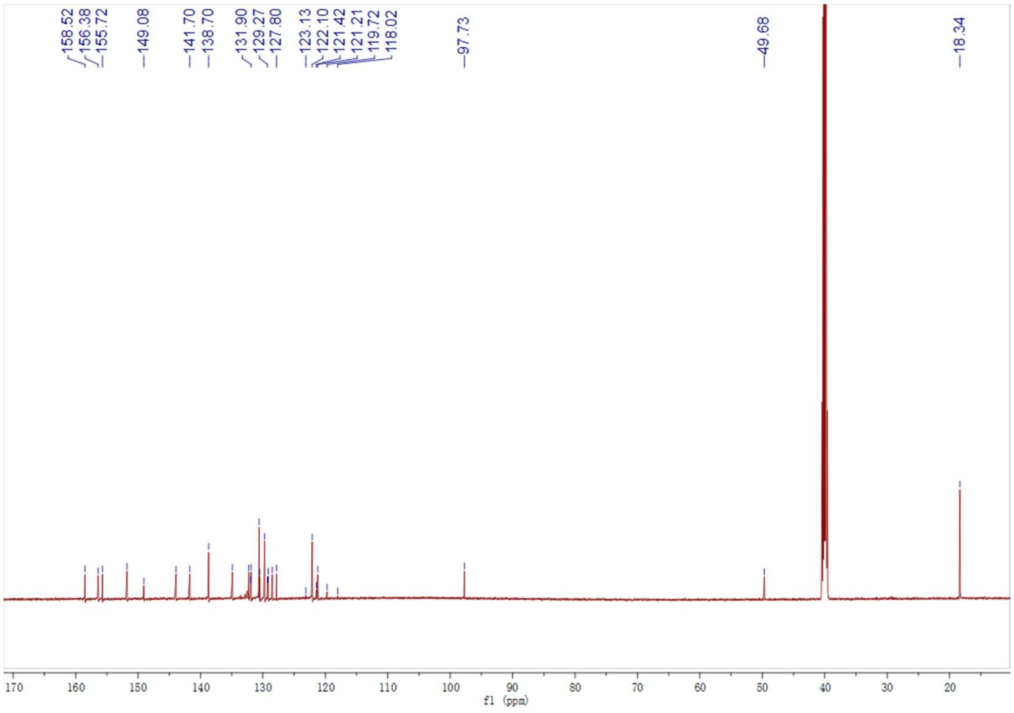

Compound 15j

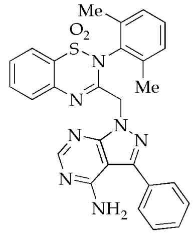

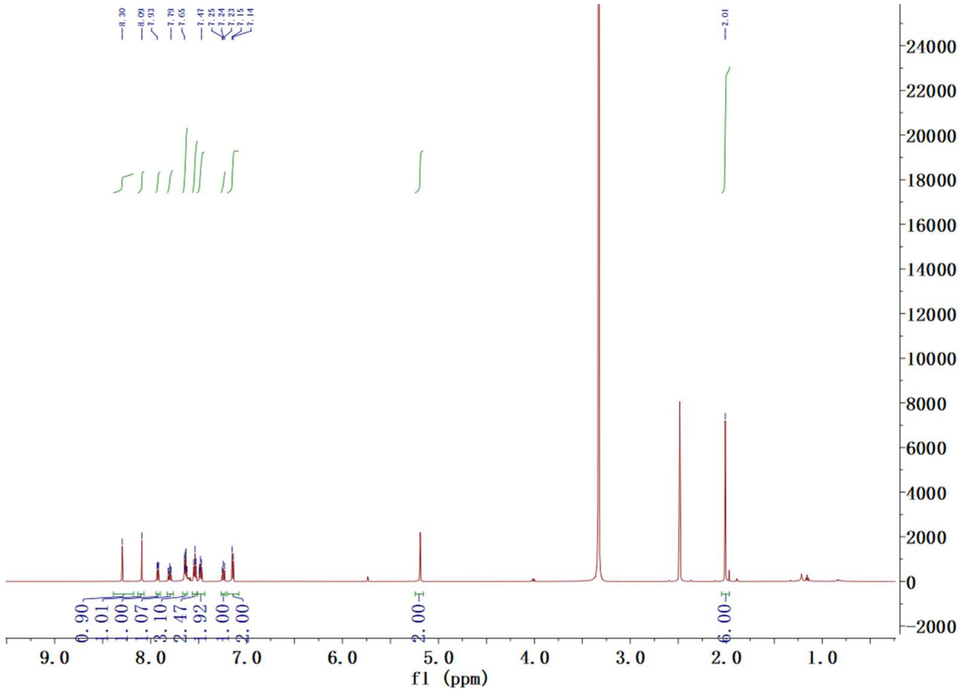

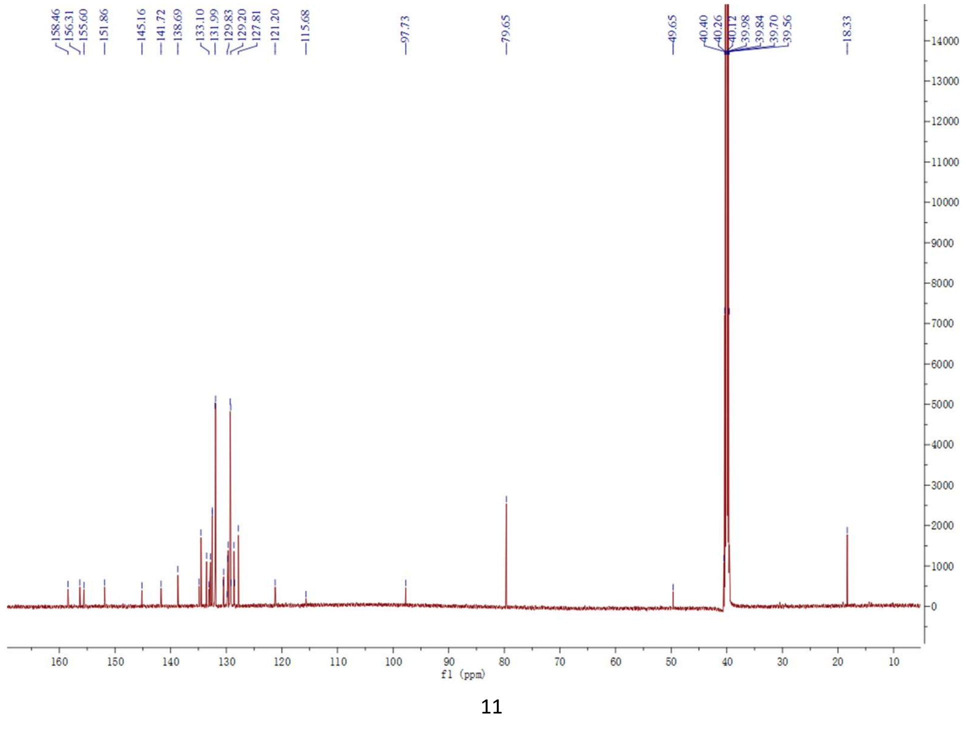

Compound 16a

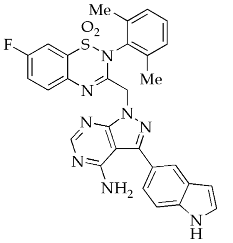

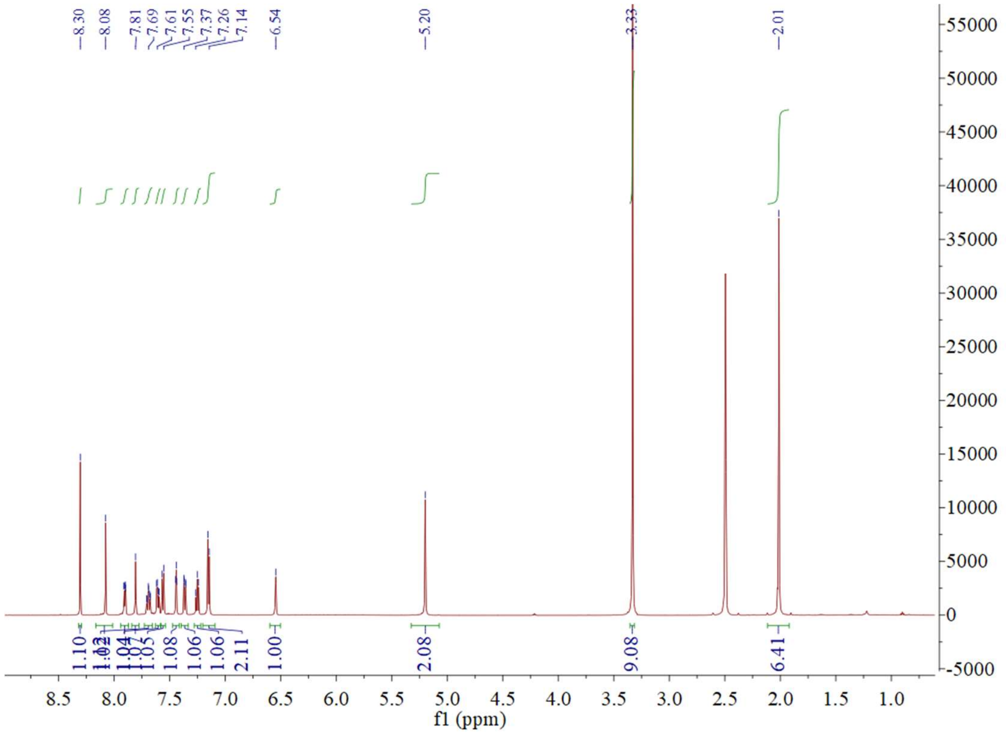

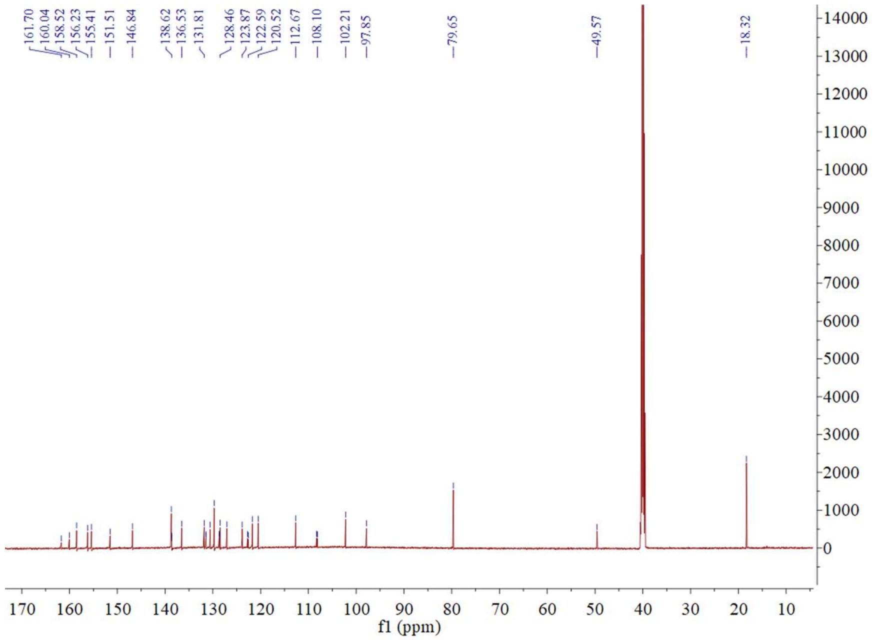

Compound 16b

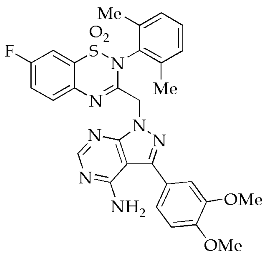

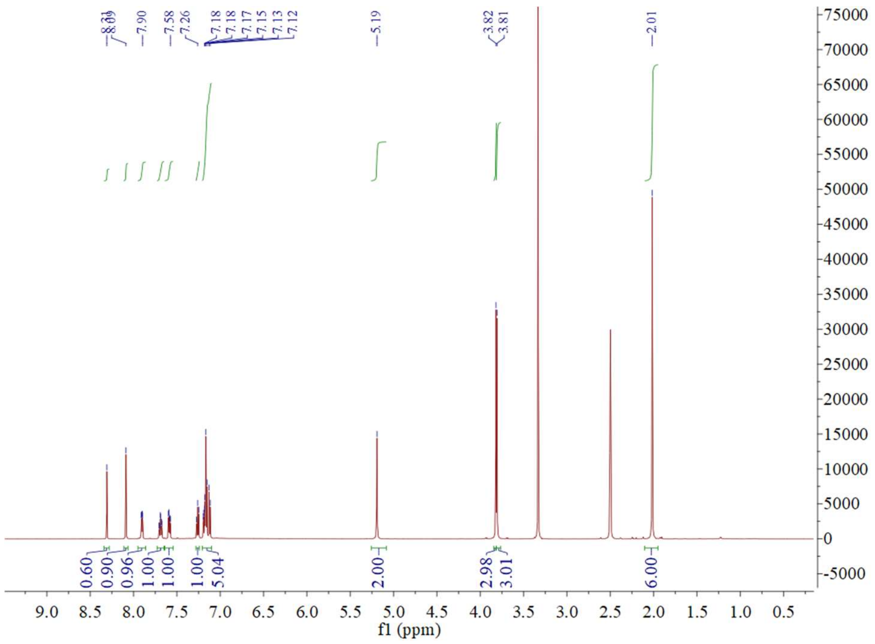

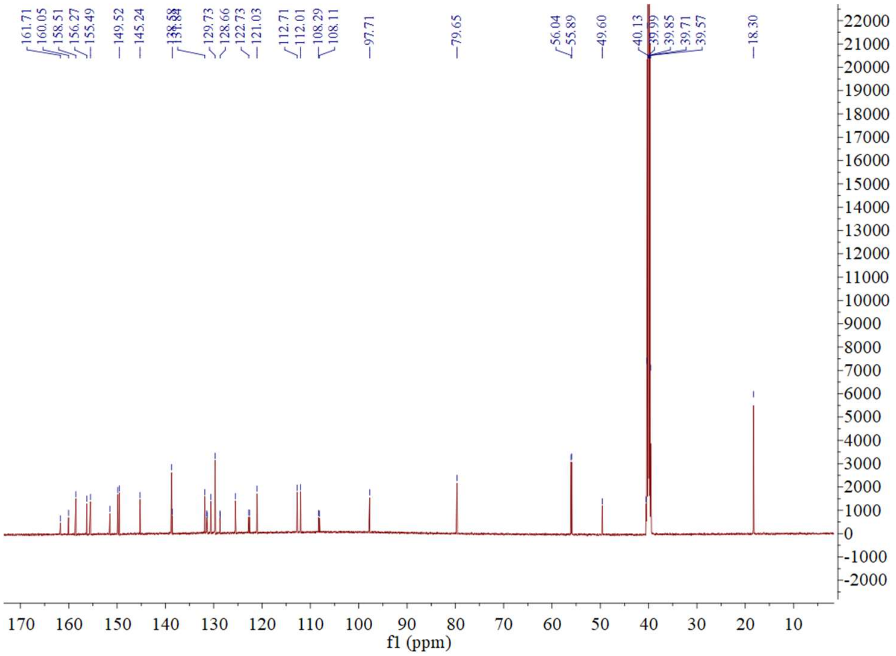

Compound 16c

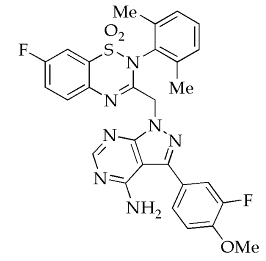

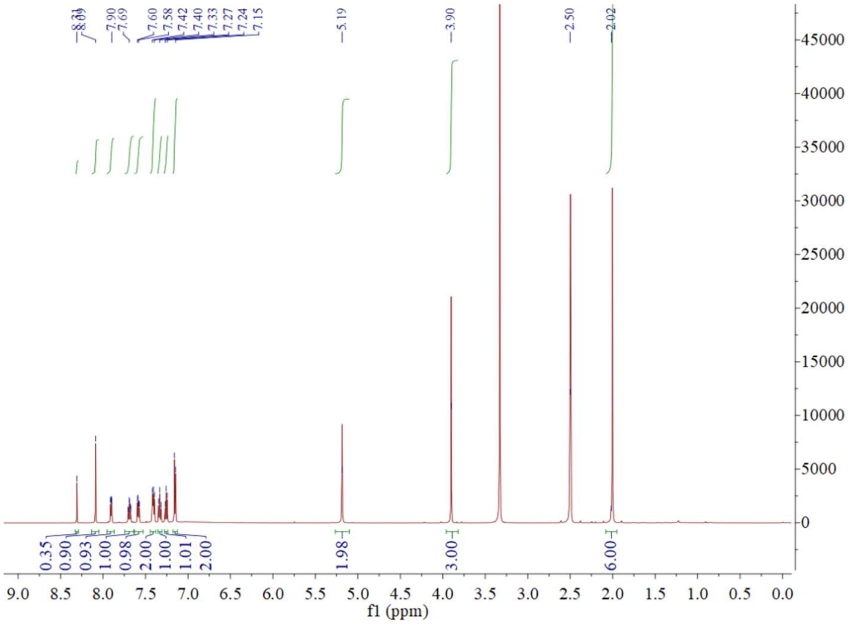

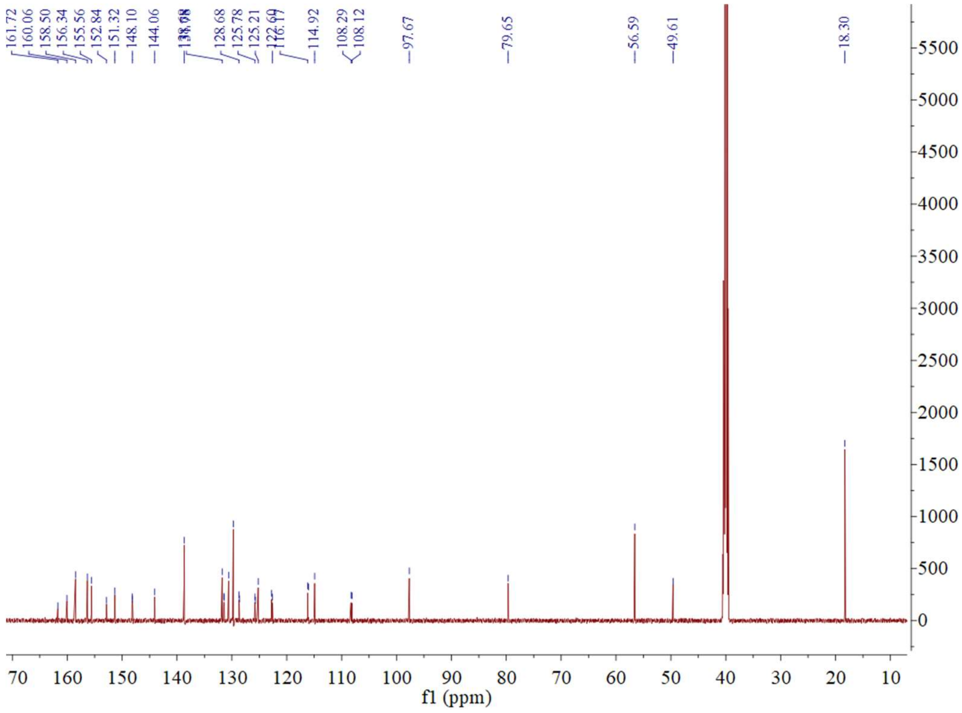

Compound 16d

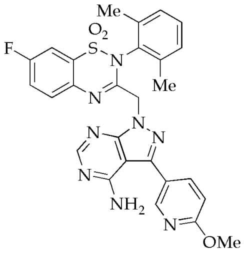

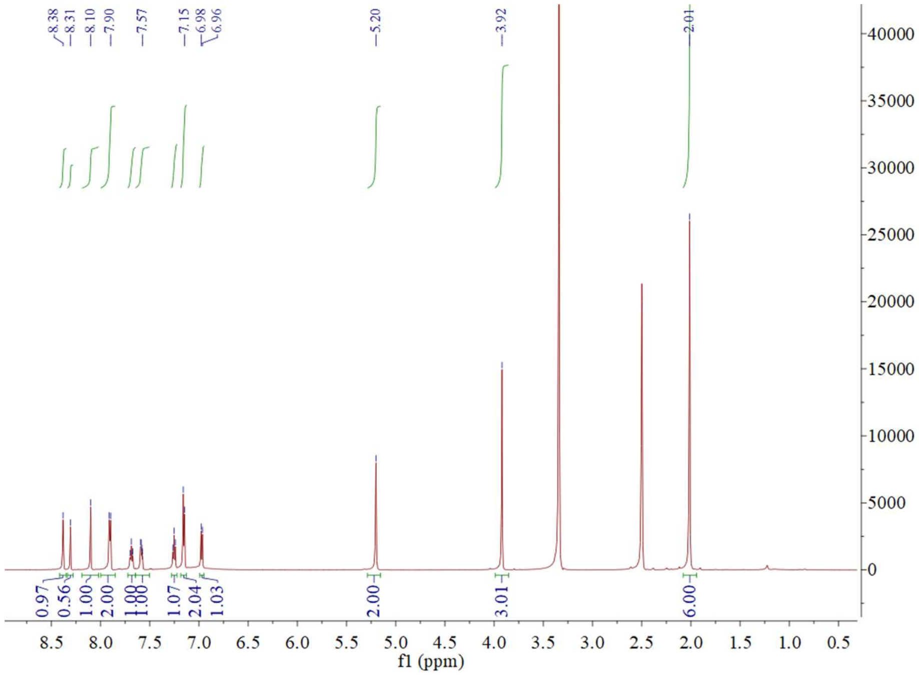

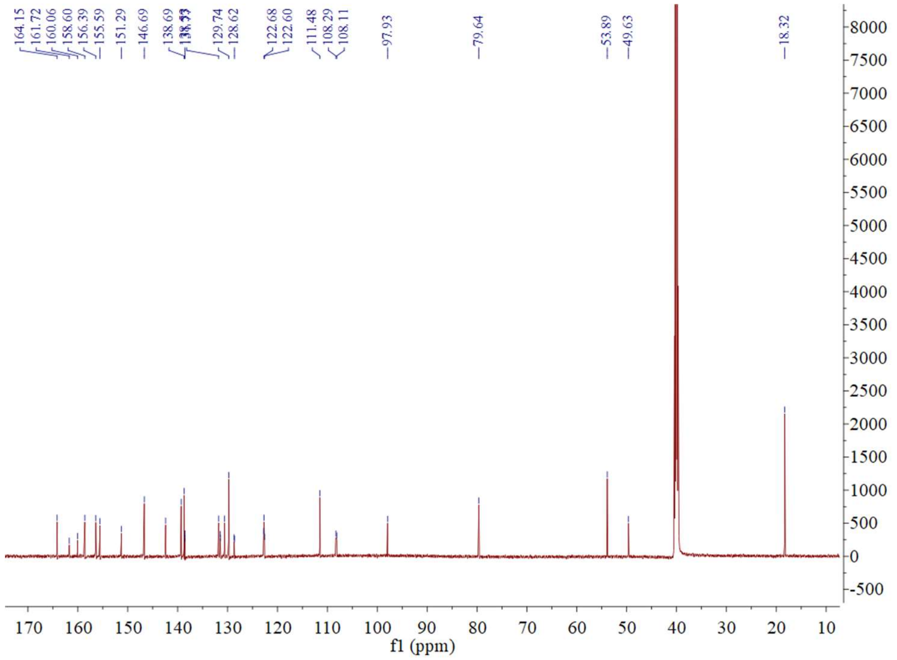

Supplement: Supplementary file 1 [file molecules-24-04299-s001.pdf]
